# Simultaneous interlayer and intralayer space control in two-dimensional metal−organic frameworks for acetylene/ethylene separation

**DOI:** 10.1038/s41467-020-20101-7

**Published:** 2020-12-07

**Authors:** Jin Shen, Xin He, Tian Ke, Rajamani Krishna, Jasper M. van Baten, Rundao Chen, Zongbi Bao, Huabin Xing, Mircea Dincǎ, Zhiguo Zhang, Qiwei Yang, Qilong Ren

**Affiliations:** 1 Key Laboratory of Biomass Chemical Engineering of Ministry of Education, College of Chemical and Biological Engineering, Zhejiang University, 310027 Hangzhou, China; 2 Department of Chemistry, Massachusetts Institute of Technology, 77 Massachusetts Avenue, Cambridge, MA 02139 USA; 3 Van ‘t Hoff Institute for Molecular Sciences, University of Amsterdam, Science Park 904, 1098 XH Amsterdam, The Netherlands

**Keywords:** Chemical engineering, Metal-organic frameworks

## Abstract

Three-dimensional metal−organic frameworks (MOFs) are cutting-edge materials in the adsorptive removal of trace gases due to the availability of abundant pores with specific chemistry. However, the development of ideal adsorbents combining high adsorption capacity with high selectivity and stability remains challenging. Here we demonstrate a strategy to design adsorbents that utilizes the tunability of interlayer and intralayer space of two-dimensional fluorinated MOFs for capturing acetylene from ethylene. Validated by X-ray diffraction and modeling, a systematic variation of linker atom oxidation state enables fine regulation of layer stacking pattern and linker conformation, which affords a strong interlayer trapping of molecules along with cooperative intralayer binding. The resultant robust materials (ZUL-100 and ZUL-200) exhibit benchmark capacity in the pressure range of 0.001–0.05 bar with high selectivity. Their efficiency in acetylene/ethylene separation is confirmed by breakthrough experiments, giving excellent ethylene productivities (121 mmol/g from 1/99 mixture, 99.9999%), even when cycled under moist conditions.

## Abstract (web summary)

Designing efficient adsorbents for trace gas removal remains a serious challenge. Here, the authors show promise in layered 2D metal−organic frameworks, often overlooked in favor of 3D frameworks, for separating trace acetylene from ethylene with enhanced performance and high stability.

## Introduction

The huge demand for high-purity gas products in industry has driven a significant development of adsorption technology for gas separation^1–3^. In particular, for the separation of trace gases from gas streams, adsorption is often more attractive than other techniques such as cryogenic distillation and solvent absorption in view of process economy or product purity. Ideal adsorbents should exhibit both high selectivity and large adsorption capacity for the trace component. However, conventional porous materials such as zeolites, activated carbon and resins have difficulty meeting all these requirements simultaneously^4–6^.

Metal−organic frameworks (MOFs), or porous coordination polymers (PCPs), are emerging porous solid materials in which open lattices are formed from inorganic nodes and organic linkers^7^. Owing to their inherent diversity, these materials enable precise control of pore shape, pore chemistry and pore size, thereby providing a versatile platform for separation processes^8–15^. In terms of trace gas removal, numerous studies have focused on the rational design of three-dimensional (3D) MOFs with uniform sub-nanometer pores^16–25^. Despite significant achievements, designing new materials that outperform existing benchmark adsorbents remains a formidable challenge. For example, in industrial processes for producing polymer-grade ethylene (C_2_H_4_), the design of efficient porous materials for acetylene (C_2_H_2_) separation is the key to replacing solvent absorption and catalytic hydrogenation technologies. The leading materials in capturing trace C_2_H_2_ from C_2_H_4_, including the NKMOF-1-Ni (Cu[Ni(pdt)_2_]))^26^ and the SIFSIX family^27–29^, all have a 3D coordination network structure that affords a high affinity for C_2_H_2_ (Table S13), but the separation performance still needs improvement in view of the limited productivity of high-purity C_2_H_4_, and the material with benchmark C_2_H_4_ productivity was reported to be unstable to water and even laboratory atmosphere^30–32^.

Herein, we demonstrate a strategy to improve the performance of adsorption separation that utilizes the tunability of both interlayer and intralayer space of two-dimensional (2D) fluorinated MOFs. This strategy results in record capacity of C_2_H_2_ at low pressures and record productivity of high-purity C_2_H_4_ coupled with superior stability. Compared to 3D MOFs, layered 2D MOFs often exhibit greater degree of flexibility owing to the possible layer movement or cross-linking in addition to local bond length/angle change during guest removal or accommodation, which allows control of porosity through or between layers. This feature has contributed to many interesting properties, such as self-accelerating gas adsorption^33^, switchable channels^34^, unexpected hydrolytic stability^35^, crystal-downsizing effect^36–38^, tunable optical properties^39^, and irreversible structural expansion^40^. When losing solvents, the interlayer stacking of 2D MOFs is often too close to accommodate guest molecules at low pressures, thereby leading to a limited performance in capturing trace component^41–44^. We targeted the deliberate creation of permanent interlayer space with desired size and chemistry together with optimal intralayer channel structures, through regulating the structure and supramolecular interactions of the 2D coordination network, in layered fluorinated MOF materials that have electronegative moieties as binding sites for guest molecules. The multiple binding sites in the interlayer ultramicroporous space, coupled with a synergistic intralayer uptake, create a high performance of separating trace C_2_H_2_ from C_2_H_4_ even under moist conditions for multiple cycles.

## Results

### Synthesis and characterization of MOFs

**Fig. 1:**
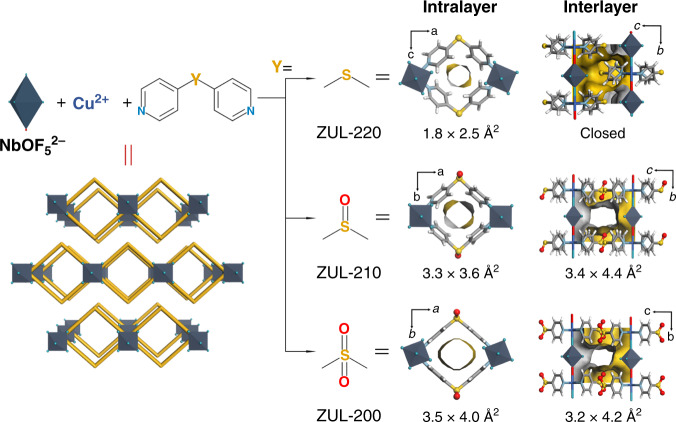
Crystal structures. X-ray crystal structures of activated ZUL-220, ZUL-210, and ZUL-200 (Color code: F, teal; Nb, plain blue; C, gray; H, white; N, sky blue; S, yellow; O, red; Cu, blue).

**Fig. 2:**
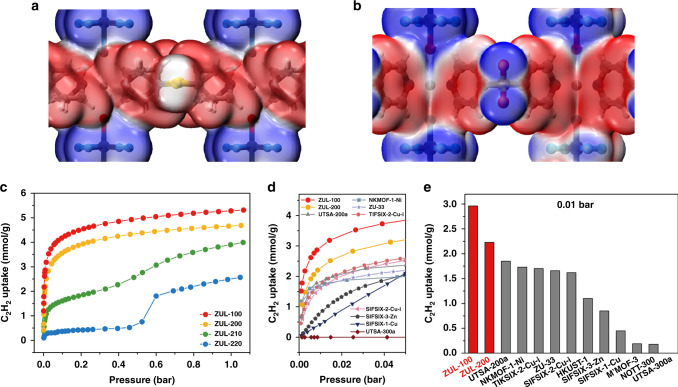
Surface electrostatic potential and C_2_H_2_ adsorption isotherms of the MOFs. **a**, **b** The local surface electrostatic potential of the 2D network in (**a**) ZUL-220 and (**b**) ZUL-200 mapped onto the 0.001 a.u. density isosurface with a scale spanning −0.03 a.u. (blue) through 0 (white) to 0.03 a.u. (red). **c** The C_2_H_2_ adsorption isotherms at 298 K for ZUL-100, ZUL-200, ZUL-210, and ZUL-220. **d** Comparison of C_2_H_2_ uptake (0–0.05 bar) among representative MOFs at 298 K. **e** Comparison of C_2_H_2_ uptake (0.01 bar, 298 K) among representative MOFs.

We synthesized a series of 2D MOFs with layered structures, [Cu(4,4’-dipyridylsulfone)_2_(NbOF_5_)] (termed as ZUL-200), [Cu(4,4’-dipyridylsulfoxide)_2_(NbOF_5_)] (ZUL-210), [Cu(4,4’-dipyridylsulfide)_2_(NbOF_5_)] (ZUL-220), using NbOF_5_^2−^ and three allied organic ligands differing in the sulfur oxidation state as mixed linkers (Fig. 1). Crystal structures were determined by single-crystal X-ray diffraction studies for both the as-synthesized and the activated samples. In all cases, quasi-one-dimensional (1D) chains of organic ligands and Cu (II) centers are bridged by NbOF_5_^2−^ in a direction perpendicular to the chain plane to form 2D coordination networks containing 1D channels, and the independent nets stack with each other via supramolecular interactions to form a layered structure. The as-synthesized samples of these three layered 2D MOFs have almost the same interlayer stacking patterns and similar linker conformations (Fig. S7). However, after activation, significant differences are observed, showing the ability of altering the oxidation state of sulfur atom in organic ligand to simultaneously control the interlayer and intralayer space. In sulfide-based material ZUL-220, adjacent 2D nets show a staggered stacking and occlusion through close S···F interactions (Figs. S8–S10), resulting in no open space between layers to accommodate gas molecules. In contrast, in sulfoxide/sulfone-based ZUL-200 and ZUL-210, the 2D nets stack in an eclipsed fashion via interactions between sulfoxide/sulfone moieties and pyridine rings, which enlarges the interlayer space and creates interlayer ultramicroporous channels along the *a* axis. The aperture size of interlayer channel is 3.4 × 4.4 Å^2^ in ZUL-210 (excluding van der Waals radii) and reduces to 3.2 × 4.2 Å^2^ in ZUL-200, both of which match well with the molecule size of C_2_H_2_ (ca. 3.3 Å). We attribute the difference in stacking patterns to the impact of oxidation on the surface electrostatic potentials of 2D nets. The sulfide moiety in ZUL-220 has a weakly positive electrostatic potential, thus it should be repelled by the positive Cu center and pyridine ring but attracted by the negative anion from adjacent nets (Fig. 2a). However, the oxidation of sulfur changes the local electrostatic potential from positive to negative, making the sulfoxide/sulfone moiety in ZUL-200 and ZUL-210 repelled by the anion but attracted by Cu and pyridine ring (Fig. 2b). This is similar to the chemistry of layered silicates that change in electrostatic properties can affect layer displacement^45^. On the other hand, the oxidization of sulfur atom also alters its conjugation with the pyridyl group and then the conformation of pyridine rings (Figs. S8–S10), making the intralayer channel gradually changes from a flexible structure to a rigid structure. In ZUL-220, the pyridine ring exhibited a rotation from 32.55° to 43.99° (between pyridine ring and Nb-Cu-N plane) upon activation, generating a very small window of 1.8 × 2.5 Å^2^ that may hinder the uptake of C_2_H_2_ at low pressures. In ZUL-210, the rotation weakens to 23.88°, leading to an expanded window of 3.3 × 3.6 Å^2^. In ZUL-200, the pyridine ring remains perpendicular to the plane before and after activation without rotation, thereby further enlarging the window size (3.5 × 4.0 Å^2^) to facilitate gas capture. The interlayer and intralayer channels are intersected with each other in the latter two materials. To further regulate the layered structure, we replaced the NbOF_5_^2−^ anions in ZUL-200 with smaller hexafluorotitanate (TiF_6_^2−^). The resultant material ZUL-100 ([Cu(4,4’-dipyridylsulfone)_2_(TiF_6_)]) is isostructural to ZUL-200, in which the intralayer channel size hardly changed but the interlayer channels contracted along the *c* axis to 3.1 Å (vs. 3.3 Å in ZUL-200, Fig. S11). The specific surface areas determined from the 77 K N_2_ adsorption isotherms were 548, 471, and 354 m^2^/g for ZUL-100, ZUL-200, ZUL-210, respectively, higher than that of ZUL-220 (326 m^2^/g determined from the 195 K CO_2_ adsorption isotherm, Figs. S14–S18). Thermogravimetric analysis revealed that these materials were stable until 473 K (Fig. S19). Moreover, no loss of crystallinity was observed for ZUL-100 and ZUL-200 during the exposure to air or soaking in water and even strong acid (pH = 1) or alkali (pH = 12) solutions, suggesting that these materials are rather robust (Figs. S5-S6).

### Single-component equilibrium adsorption isotherms

Single-component equilibrium adsorption isotherms for C_2_H_2_ were collected at ambient conditions as shown in Fig. 2c. Both isotherm shape and adsorption capacity largely depend on the sulfur oxidation state in the organic ligand. ZUL-220 exhibits a clear multi-step isotherm with very low C_2_H_2_ uptake (<0.55 mmol/g) below 0.5 bar, followed by a significant uptake step typically attributed to structural flexibility. A similar flexible behavior, albeit less obvious, is observed in ZUL-210, whose isotherm also exhibits an inflexion point at approximately 0.5 bar. In contrast, the rigid sulfone-based materials ZUL-200 and ZUL-100 show steep isotherms at very low pressures and higher adsorption capacities over ZUL-210 and ZUL-220, demonstrating a good affinity for C_2_H_2_. To our knowledge, ZUL-100 adsorbs more C_2_H_2_ than other benchmark materials in the industrially relevant pressure range of 0.001–0.05 bar, which covers the diversity of C_2_H_2_ content in industrial feed gas (0.3–1%) (Fig. 2d, Table S13). At 0.01 bar, the C_2_H_2_ uptake of ZUL-100 is 2.96 mmol/g, at least 1.6 times as large as the values of previous benchmarks UTSA-200a (1.85 mmol/g)^30^, NKMOF-1-Ni (1.73 mmol/g)^26^, TIFSIX-2-Cu-i (1.70 mmol/g)^27^ and ZU-33 (1.66 mmol/g)^29^. At 0.003 bar, the C_2_H_2_ uptake of ZUL-100 is 2.25 mmol/g, again surpassing that of the previous benchmark (NKMOF-1-Ni, 1.50 mmol/g)^26^ (Fig. 2d). The volumetric C_2_H_2_ uptakes of ZUL-100 at low pressures are also record high, e.g., 4.01 mmol/ml at 0.01 bar and 298 K (Table S12). Although ZUL-200 has lower C_2_H_2_ uptake than ZUL-100, it still outperforms existing benchmarks in the range 0.003–0.05 bar. As detailed below, we attribute the high performance of these materials to their optimal interlayer and intralayer structures.

### DFT-D calculation and guest-loaded single crystal XRD

Modeling studies using first-principles dispersion-corrected density functional theory (DFT-D) provided insight into the structure change of the 2D fluorinated MOFs. We used the solvent-omitted as-synthesized crystal structures as the hypothetical activated model where no movement of the layer and the linker rotation has been allowed. The structures were optimized using the DFT calculation, with all atomic positions and unit cell parameters allowed to vary. The difference in the calculated energies between the practical activated model and the hypothetical activated model should be, to a first approximation, a measure of the energy needed to move the layer and rotate the linker. The practical activated ZUL-220 model was found to be 113 kJ/mol lower in energy than the hypothetical activated ZUL-220 model, and the practical model was 3.2 kJ/mol and 0.53 kJ/mol lower than the hypothetical model for ZUL-210 and ZUL-200, respectively. Of cause, this fairly simple analysis does not take into account the energy barriers to the structural changes on gas accommodation, which will increase the energy required for the transformation, especially of ZUL-220, further limiting the gas accommodation at low pressures. This is consistent with the results of experimental adsorption isotherms, supporting that ZUL-220 and ZUL-210 are relatively flexible while ZUL-200 is rigid, and ZUL-220 is more flexible than ZUL-210. In addition, literature works^46^ have shown that the energy barrier for the rotation of aromatic ring in different MOFs is lower than 25 kJ/mol, so the structural change of ZUL-220 is considered to be mainly caused by the layer motion rather than linker rotation.

**Fig. 3:**
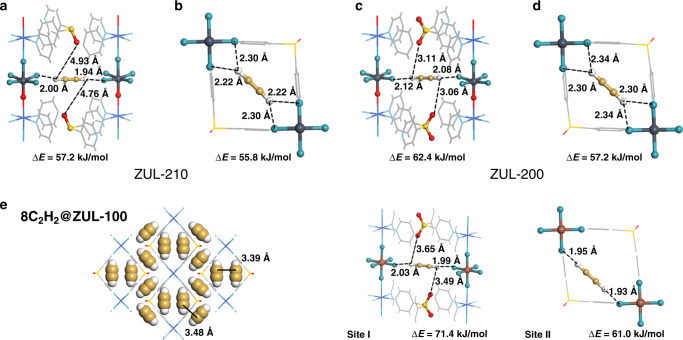
DFT-D calculated C_2_H_2_ binding modes. **a**, **b** The DFT-D calculated C_2_H_2_ binding mode in ZUL-210. **c**, **d** The DFT-D calculated C_2_H_2_ binding mode in ZUL-200. **e** View of the binding modes for adsorbed C_2_H_2_ molecules in ZUL-100 (eight C_2_H_2_ per unit cell) (Color code: F, teal; Nb, plain blue; Ti, brown; C (in framework), gray; H, white; N, sky blue; S, yellow; O, red; Cu, blue; C (in C_2_H_2_), golden).

DFT-D calculation further provided insight into the adsorption behavior of the 2D fluorinated MOFs. In ZUL-220, because of closed interlayer and intralayer space, a C_2_H_2_ molecule cannot be trapped at low pressure. In ZUL-210, C_2_H_2_ can be bound in the intralayer channel, with four F atoms from two diagonal NbOF_5_^2−^ of the same pseudo-cubic cavity, but an energy favorable binding site emerges in the interlayer channel, at which one C_2_H_2_ molecule is bound by two F atoms from different 2D networks through shorter C–H···F hydrogen bonds (Fig. 3a, b). The calculated static adsorption energy at this site is 57.2 kJ/mol, slightly stronger than the binding at the intralayer site (55.8 kJ/mol). In ZUL-200, both intralayer and interlayer binding sites exist as well. However, when trapped at the interlayer site, a C_2_H_2_ molecule is bound both by two F atoms and by two O atoms from different 2D networks through considerable van der Waals interactions (Fig. 3c, d). In total, two F atoms and two O atoms surround the C_2_H_2_ molecule from different directions, altogether contributing to a static adsorption energy of 62.4 kJ/mol. This energy is not only stronger than the intralayer binding in this material (57.2 kJ/mol) and the interlayer binding in ZUL-210, but also is higher than that reported for other benchmark materials^26–30^. When C_2_H_2_ is trapped in ZUL-100 with the same organic linker but TiF_6_^2−^ anion, both the interlayer binding and intralayer binding of C_2_H_2_ molecule are further enhanced, with static energies of 71.4 kJ/mol and 61.0 kJ/mol, respectively (Fig. 3e). The presence of abundant binding sites with high energies contributes to the extraordinary uptake of C_2_H_2_ in ZUL-100 and ZUL-200 at low pressures.

The cooperation of interlayer and intralayer binding sites in C_2_H_2_ adsorption are demonstrated by DFT-D calculations when more guest molecules are involved. When six C_2_H_2_ molecules are trapped in each unit cell of ZUL-200, which corresponds to the experimental adsorption capacity at 1 bar (6.6 molecules per unit cell), two molecules are bound at the primary sites between layers, and the other one at the secondary site in the intralayer channel (Fig. S32). Similarly, when eight C_2_H_2_ molecules are trapped in each unit cell of ZUL-100, representing the adsorption capacity at 1 bar (7.1 per unit cell), two of them are bound at the interlayer sites and the other two at the intralayer sites, showing a highly efficient packing of C_2_H_2_ molecules in the layered structure with an average energy of 59.4 kJ/mol for each C_2_H_2_ (Fig. 3e). The distance between the two C_2_H_2_ molecules in the same cavity is 3.3 Å, implying a guest-guest interaction through π–π overlapping that is synergistic with the host-guest C–H···F interactions and likely contributes to the strong adsorption, making the C_2_H_2_ uptake at 1 bar higher than those of reference materials having a 3D ultramicroporous coordination network (Table S13).

**Fig. 4:**
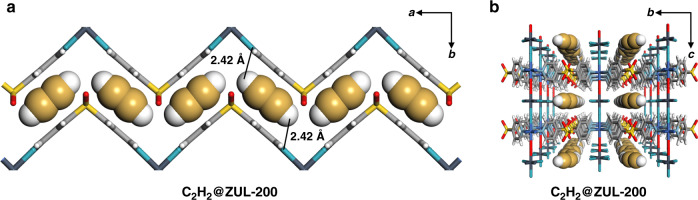
The single crystal structure of C_2_H_2_@ZUL-200. **a** The single crystal structure of ZUL-200 under C_2_H_2_ (C_2_H_2_@ZUL-200) at ultralow pressure viewed along the *c* axis. C_2_H_2_ molecules are trapped in interlayer space with dual C–H···F hydrogen bonds (2.42 Å). **b** The single crystal structure of C_2_H_2_@ZUL-200 viewed along the *a* axis (Color code: F, teal; Nb, plain blue; C (in framework), gray; H, white; N, sky blue; S, yellow; O, red; Cu, blue; C (in C_2_H_2_), golden).

The crystal structure of ZUL-200 under C_2_H_2_ (C_2_H_2_@ZUL-200) at ultralow pressure was determined to verify the DFT-D-computed binding sites. Activated ZUL-200 single crystals suitable for X-ray diffraction were filled with C_2_H_2_ at room temperature. “C_2_H_2_ zigzags” along the *a* axis was observed in the interlayer space, with each C_2_H_2_ molecule forming dual C–H···F hydrogen bonds (2.42 Å) with two NbOF_5_^2−^ anions from adjacent layers (Fig. 4, S29). This is in agreement with the strongest binding site determined by DFT-D calculation, supporting the significance of interlayer space control on the capture of trace C_2_H_2_. The experimental isosteric enthalpy of adsorption (*Q*_st_) was calculated for ZUL-200 and ZUL-100 (Figs. S27–S28). The zero-coverage *Q*_st_ for C_2_H_2_ on ZUL-100 and ZUL-200 is 65.3 kJ/mol and 57.6 kJ/mol, respectively, notably higher than the *Q*_st_ for C_2_H_4_ (<40 kJ/mol).

### Selectivity and co-adsorption simulations

**Fig. 5:**
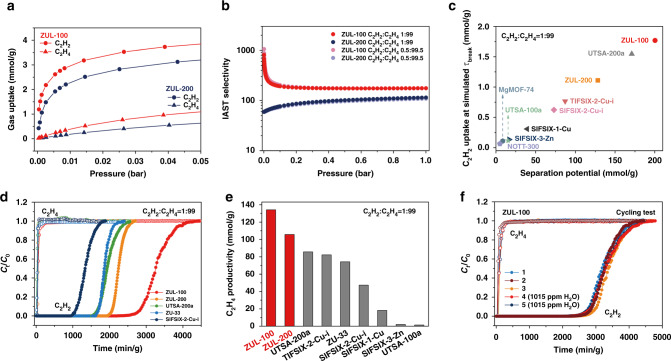
C_2_H_2_ and C_2_H_4_ adsorption isotherms, IAST calculations, simulated and experimental column breakthrough results. **a** C_2_H_2_ and C_2_H_4_ adsorption isotherms of ZUL-100 and ZUL-200 at 298 K. **b** The IAST selectivities of ZUL-100 and ZUL-200 for C_2_H_2_/C_2_H_4_ mixtures. **c** Plots of the captured C_2_H_2_ amount from the simulated column breakthrough as a function of separation potential from IAST calculations for C_2_H_2_/C_2_H_4_ (1/99) separation. **d** Experimental column breakthrough curves for C_2_H_2_/C_2_H_4_ (1/99) mixture with ZUL-100, ZUL-200, UTSA-200a, ZU-33, and SIFSIX-2-Cu-i at 298 K and 1 bar. **e** Experimental C_2_H_4_ productivity (C_2_H_2_ below 40 ppm) from C_2_H_2_/C_2_H_4_ (1/99) mixture through fixed bed adsorbers packed with ZUL-100, ZUL-200 and other materials at 298 K and 1 bar. **f** The recyclability of ZUL-100 under multiple mixed-gas (including two cycles under moist feed stream) column breakthrough tests.

The selectivity of C_2_H_2_ to C_2_H_4_ is also important in order to obtain ultrahigh purity C_2_H_4_. Due to the weaker H-bond acidity of C_2_H_4_ relative to C_2_H_2_, the C_2_H_4_ uptake of ZUL-100 and ZUL-200 is much lower than the C_2_H_2_ uptake (Fig. 5a). DFT-D calculations show that C_2_H_4_ molecules can be adsorbed by both interlayer and intralayer channels, but the static binding energy (41.5 kJ/mol) (Figs. S33–S34) is significantly lower for C_2_H_4_ than for C_2_H_2_. Considering the concentration range of C_2_H_2_ in C_2_H_4_ feed gas is 0.3–1%, the selectivities of 0.5:99.5 and 1:99 mixtures are calculated (Fig. 5b) with the ideal adsorbed solution theory (IAST). At 1 bar, C_2_H_2_:C_2_H_4_ = 1:99, the selectivity is as high as 175 for ZUL-100, more than three times as high as all materials except the moisture-sensitive materials UTSA-200a (SIFSIX-14-Cu-i) and ZU-33 (GeFSIX-14-Cu-i) (Table S13). The IAST calculated C_2_H_2_ capacity for C_2_H_2_:C_2_H_4_ = 1:99 mixture co-adsorption in ZUL-100 are the highest of reported MOFs, e.g., the capacity at 0.01 bar is 2.03 mmol/g (Fig. S35), even higher than the single-component C_2_H_2_ uptake at 0.01 bar in previous benchmarks. Given that the productivity of high-purity C_2_H_4_ will mainly be limited by the trace C_2_H_2_ uptake capacity when the selectivity is already at a very high level, the ultrahigh capacity and selectivity of ZUL-100 and ZUL-200 for trace C_2_H_2_ make it possible to obtain a superior C_2_H_4_ productivity although the IAST selectivity is not the highest. Recently, Krishna^47,48^ introduced a new combined metric, separation potential (Δ*q*), which represents the maximum number of moles of the less strongly adsorbed species that can be recovered in the gas phase per gram of adsorbent in the fixed bed, taking into account the effect of both selectivity and uptake capacity at the same time and matching to the real processes better, for evaluating the separation performance in fixed bed adsorbers. For separating C_2_H_2_:C_2_H_4_ = 1:99 mixture in a fixed bed at room temperature, ZUL-100 exhibits the record separation potential (200 mmol/g) (Fig. 5c). Although the separation potential of ZUL-200 is somewhat inferior to ZUL-100, it is also superior to other robust benchmark materials.

It’s worth mentioning that configurational-bias Monte Carlo (CBMC) simulations have also been tested for this system. The unary isotherm for C_2_H_4_ was reproduced very well by an united atom model, but a notable deviation exists between simulation and experiments for the unary isotherm for C_2_H_2_ even if diverse simulation models were employed, probably due to the structural particularity of these layered materials and the limitations of the Lorentz–Berthelot mixing rule (Fig. S36). Nevertheless, the reliability of IAST calculation on estimating the 1/99 mixture adsorption equilibria has been supported by CBMC simulations with scaled parameters for C_2_H_2_ and unscaled parameters for C_2_H_4_ (Figs. S37–38), implying that ZUL-100 and ZUL-200 can have great C_2_H_2_/C_2_H_4_ separation performance.

### Breakthrough separation

The feasibility of using ZUL-100 and ZUL-200 in a fixed bed at given operating conditions for the separation of C_2_H_2_/C_2_H_4_ (1/99, v/v) mixture at room temperature was investigated by transient breakthrough simulations. For ZUL-100, the C_2_H_4_ was immediately eluted through the adsorption bed as a high-purity grade gas (Fig. S39), whereas C_2_H_2_ was retained in the packed column with the longest breakthrough time τ_break_ (8860), revealing high C_2_H_2_ capacity (1.77 mmol/g, at τ_break_) and C_2_H_4_ productivity (4.00 L/g) (Fig. 5c, Fig. S40). Likewise, ZUL-200 shows high τ_break_ (6032) and C_2_H_2_ capacity (1.11 mmol/g), indicating a promising separation performance.

We further examined the actual performances of ZUL-100 and ZUL-200 for C_2_H_2_/C_2_H_4_ (1/99, v/v) mixture separation at room temperature by experimental breakthrough tests. Indeed, the separation of C_2_H_2_/C_2_H_4_ mixture was efficient, in agreement with the simulated breakthrough results (Fig. S41). As shown in Fig. 5d, for ZUL-100, C_2_H_4_ was immediately eluted from the packed column as high-purity grade gas, whereas C_2_H_2_ can be retained on the column for more than 2200 min/g with an outlet concentration below 1 ppm. This C_2_H_2_ breakthrough time is 1.7 times as long as that observed for UTSA-200a (ca. 1300 min/g)^30^ and also far exceeds that of ZU-33 (1570 min/g)^29^. The C_2_H_2_ breakthrough time in ZUL-200 (1920 min/g) is shorter than that in ZUL-100, but still longer than all the reference materials. Although the transient breakthrough simulation implies a slower C_2_H_2_ breakthrough in UTSA-200a than in ZUL-200, the experimental C_2_H_2_ uptake in breakthrough tests of UTSA-200a determined by two independent groups is notably smaller than the simulation value (Fig. S41)^30,32^. The purity of C_2_H_4_ in the outlet effluent was analyzed to be >99.9999% with the C_2_H_2_ concentration less than 1 ppm, which satisfies the quality standards of polymerization grade C_2_H_4_ (C_2_H_4_ > 99.95%, C_2_H_2_ < 5 ppm)^49,50^. During the breakthrough process, the C_2_H_2_ uptake and C_2_H_4_ productivity (>99.9999% purity) from 1:99 mixtures of C_2_H_2_:C_2_H_4_ gave record values of 1.92 mmol/g, 121.2 mmol/g for ZUL-100 and 1.26 mmol/g, 103.6 mmol/g for ZUL-200, respectively. The C_2_H_2_ breakthrough time with an outlet concentration below 40 ppm, which is employed by most related reference to calculate C_2_H_4_ productivity, was 2546 min/g for ZUL-100 and 1958 min/g for ZUL-200, giving a C_2_H_4_ productivity of 134.1 mmol/g and 105.7 mmol/g, respectively. These are significantly higher than the C_2_H_2_ uptake and C_2_H_4_ productivity (C_2_H_2_ below 40 ppm) reported for reference materials, e.g., 1.18 mmol/g and 85.7 mmol/g for UTSA-200a^30^_,_ 0.83 mmol/g and 82.2 mmol/g for TIFSIX-2-Cu-i^27^ and 0.94 mmol/g and 74.2 mmol/g for ZU-33^29^ (Fig. 5d, 5e).

The feed gas streams in practical C_2_H_2_ removal unit often contain trace level of H_2_O (<5 ppm), and an ideal adsorbent must keep performance in the presence of H_2_O. Importantly, breakthrough experiments of 1:99 mixtures containing 1015 ppm H_2_O conducted with ZUL-100 showed that the presence of H_2_O has a negligible effect on the separation of C_2_H_2_ from C_2_H_4_, even after multiple cycles. ZUL-100 retains the C_2_H_2_ capacity and C_2_H_4_ productivity over 5 cycles that contain two cycles under moisture (the fourth and fifth), confirming the good recyclability of this material for C_2_H_2_/C_2_H_4_ separation (Fig. 5f).

## Discussion

This study demonstrates the considerable potential of layered 2D MOFs, often overlooked in favor of ultramicroporous 3D MOFs, for separating trace gases from gas streams. For the ZUL-series of 2D fluorinated MOFs, a simple oxidation of the sulfur atom triggers a change of the surface electrostatic potential of the 2D coordination network and the linker conformation, which further changes the supramolecular interlayer stacking pattern and structural flexibility to create permanent channels with desired size and chemistry in both interlayer and intralayer space simultaneously. This affords a strong interlayer trapping of C_2_H_2_ molecules along with cooperative intralayer binding. With good stability against air, water and heat, the sulfone-based 2D fluorinated MOFs demonstrate benchmark C_2_H_2_ uptake in the industrially relevant pressure range of 0.001–0.05 bar, ultrahigh C_2_H_2_/C_2_H_4_ selectivity, and record high-purity C_2_H_4_ productivity even when cycled under moist feed stream. This is encouraging to pursue further research in engineering issues such as material shaping, large-scale synthesis and process optimization. In addition to setting a benchmark for C_2_H_2_/C_2_H_4_ separation, the strategy described in this study demonstrates a new crystal engineering approach for the synthesis of new porous materials that may enable other trace gas capture and separation.

## Methods

### Materials

All starting materials and solvents were commercially available and used without further purification. Ammonium hexafluorotitanate ((NH_4_)_2_TiF_6_, 98%, Aldrich), copper(II) tetrafluoroborate hydrate (Cu(BF_4_)_2_•*x*H_2_O, 98%, Aldrich), CuNbOF_5_(98%, Sychemlab), 4,4’-dipyridylsulfone (C_10_H_8_N_2_O_2_S, 98%, Sychemlab), 4,4’-dipyridylsulfoxide (C_10_H_8_N_2_OS, 98%, Sychemlab), 4,4’-dipyridylsulfide (C_10_H_8_N_2_S, 99%, TCI), methanol (CH_3_OH, anhydrous, 99%, Sinopharm), *N*,*N*-Dimethylformamide (DMF, C_3_H_7_NO, 99%, Sinopharm). N_2_ (99.999%), C_2_H_2_ (99%), C_2_H_4_ (99.99%), He (99.999%) and mixed gases of C_2_H_2_/C_2_H_4_ = 1/99 were purchased from JinGong Company. Mixed gases of 1015 ppm H_2_O, 1% C_2_H_2_ and 98.9% C_2_H_4_ were purchased from Shanghai Wetry Standard Reference Gas Analytical Technology Co. LTD (China).

### Synthesis of ZUL-100 (Cu(4,4’-dipyridylsulfone)_2_TiF_6_)_n_

A methanol solution (10 mL) of 4,4’-dipyridylsulfone (216 mg, 1 mmol) was slowly dropped into an aqueous solution (10 mL) of (NH_4_)_2_TiF_6_ (99 mg, 0.5 mmol) and Cu(BF_4_)_2_•*x*H_2_O (118.5 mg, 0.5 mmol). Then the mixture was under stirring for 24 h at room temperature. The obtained purple powder was filtered, then washed with methanol, and was exchanged with methanol for 3 days (260.6 mg, 78% yield based on Cu).

### Synthesis of ZUL-200 (Cu(4,4’-dipyridylsulfone)_2_NbOF_5_)_n_

A methanol solution (4 mL) of 4,4’-dipyridylsulfone (43.2 mg, 0.2 mmol) was carefully layered onto an DMF (10 mL) of CuNbOF_5_ (26.7 mg, 0.1 mmol). Crystals of ZUL-200 were obtained after 5 days. Then crystals were washed with methanol, and were exchanged with methanol for 3 days. A direct mixing method was used to produce large amount of powder samples. A methanol solution (10 mL) of 4,4’-dipyridylsulfone (216 mg, 1 mmol) was slowly dropped into an aqueous solution (10 mL) of CuNbOF_5_ (133.5 mg, 0.5 mmol). Then the mixture was under stirring for 24 h at room temperature (294.8 mg, 84% yield based on Cu).

### Synthesis of ZUL-210 (Cu(4,4’-dipyridylsulfoxide)_2_NbOF_5_)_n_

A methanol solution (4 mL) of 4,4’-dipyridylsulfoxide (40.0 mg, 0.2 mmol) was carefully layered onto an DMF (10 mL) of CuNbOF_5_ (26.7 mg, 0.1 mmol). Crystals of ZUL-210 were obtained after 5 days. Then crystals were washed with methanol, and were exchanged with methanol for 3 days (38.6 mg, 58% yield based on Cu).

### Synthesis of ZUL-220 (Cu(4,4’-dipyridylsulfide)_2_NbOF_5_)_n_

A methanol solution (4 mL) of 4,4’-dipyridylsulfide (37.7 mg, 0.2 mmol) was carefully layered onto an DMF (10 mL) of CuNbOF_5_ (26.7 mg, 0.1 mmol). Crystals of ZUL-220 were obtained after 5 days. Then crystals were washed with methanol, and were exchanged with methanol for 3 days (33.5 mg, 52% yield based on Cu).

### X-ray diffraction structure analysis

Powder X-ray diffraction (PXRD) data were collected on a SHIMADZU XRD-6000 diffractometer (Cu Kαλ = 1.540598 Ǻ) with an operating power of 40 kV, 30 mA. The collected range of 2θ is 5° to 50°.

### Single crystal X-ray diffraction data

Crystal data for the as-synthesized samples of ZUL-100, ZUL-200, ZUL-210, ZUL-220 and the activated samples of ZUL-200, ZUL-210, ZUL-220 and the C_2_H_2_-loaded sample C_2_H_2_@ZUL-200 were collected at 123(2) K on a BrukerAXS D8 VENTURE diffractometer equipped with a PHOTON II detector. Indexing was performed using APEX 3. Data integration and reduction were completed using SaintPlus 6.01. Absorption correction was performed by multi-scan method implemented in SADABS. The space group was determined using XPREP implemented in APEX 3. The structure was solved with SHELXS-97 (direct methods) and refined on F^2^ (nonlinear least-squares method) with SHELXL-97 contained in APEX 3, WinGX v1.70.01, and OLEX2 v1.1.5 program packages. All non-hydrogen atoms were refined anisotropically. The contribution of disordered solvent was treated as diffuse using the Squeeze routine implemented in Platon. The crystal data are summarized in Tables S1–S8.

### Thermal gravimetric analysis

The thermal gravimetric analysis was performed on Pyris 1 TGA. Experiments were going on a platinum pan under nitrogen flow with a heating rate of 10 °C/min from 50 °C to 700 °C.

### Gas adsorption measurement

ZUL-100, ZUL-200, ZUL-210 and ZUL-220 were evacuated at room temperature for 24 h until the pressure below 5 μmHg. The measurements of C_2_H_2_ and C_2_H_4_ adsorption isotherms on activated ZUL-100, ZUL-200, ZUL-210, and ZUL-220 were collected at 273–313 K using ASAP 2460 Analyzer (Micromeritics).

### Breakthrough experiment

The breakthrough experiments were carried out in a dynamic gas breakthrough equipment^28^. All experiments were conducted using a stainless steel column (4.6 mm inner diameter × 50 mm). According to the different particle size and density of two sample powder, the weight packed in the column was: ZUL-100 (0.3125 g) and ZUL-200 (0.6873 g), respectively. The column packed with sample was firstly purged with He flow (15 mL min^−1^) for 24 h at room temperature. The binary mixed gas of C_2_H_2_/C_2_H_4_ = 1:99 (v/v) or ternary mixed gas of 1015 ppm H_2_O, 1% C_2_H_2_ and 98.9% C_2_H_4_ was then introduced at 1.25 mL min^−1^. The concentration of outlet gas from the column was monitored by gas chromatography (GC 2010 Pro, SHIMADZU) with the flame ionization detector FID. After the breakthrough experiment, the sample was regenerated with He flow (15 mL min^−1^) for 1 day.

The C_2_H_2_ concentration definition is:

1 $${\mathrm{Concentration}}\left( {{\mathrm{C}}_2{\mathrm{H}}_2} \right) = \frac{{{\mathrm{signal}}({\mathrm{C}}_2{\mathrm{H}}_2)}}{{{\mathrm{signal}}\left( {{\mathrm{C}}_2{\mathrm{H}}_2} \right) + {\mathrm{signal}}({\mathrm{C}}_2{\mathrm{H}}_4)}}$$

The C_2_H_4_ purity definition is:

2 $${\mathrm{Purity}}\left( {{\mathrm{C}}_2{\mathrm{H}}_4} \right) = \frac{{{\mathrm{signal}}({\mathrm{C}}_2{\mathrm{H}}_4)}}{{{\mathrm{signal}}\left( {{\mathrm{C}}_2{\mathrm{H}}_2} \right) + {\mathrm{signal}}({\mathrm{C}}_2{\mathrm{H}}_4)}}$$

The C_2_H_2_ uptake calculation in breakthrough experiment is defined by:

3 $$n = \frac{{\mathop {\smallint }\nolimits_0^{t_2} \left( {u_iy_{{\mathrm{C}}_2{\mathrm{H}}_2} - u_e\left( t \right)y_e(t)} \right)Adt}}{{V_m}} = \, \frac{{F \times y_{{\mathrm{C}}_2{\mathrm{H}}_2} \times \mathop {\smallint }\nolimits_0^{t_2} \left( {1 - \frac{{C\left( t \right)}}{{C_0}}} \right)dt}}{{V_m}}\\ = \, \frac{{F \times y_{{\mathrm{C}}_2{\mathrm{H}}_2} \times \left( {t_2 - \mathop {\smallint }\nolimits_0^{t_2} \frac{{C\left( t \right)}}{{C_0}}dt} \right)}}{{V_m}}$$

where *n* is the C_2_H_2_ uptake in mmol/g, *t*_2_ is the C_2_H_2_ saturation time, *u*_*e*_*(t)* is the transient linear velocity in outlet gas, *y*_*e*_*(t)* is the transient C_2_H_2_ volume fraction in outlet gas, *u*_*i*_ is the transient linear velocity in inlet gas, *F* is the inlet gas volume flow rate, $y_{{\mathrm{C}}_2{\mathrm{H}}_2}$ is the volume fraction of the C_2_H_2_ in the mixed gas, 0.01, ${\int}_0^{t_2} {\frac{{C(t)}}{{C_0}}dt}$ is the integrated area between the C_2_H_2_ breakthrough curve and the *x* axis in range of 0 *t*o *t*_2_, *C(t)* is the detected C_2_H_2_ concentration in the outlet gas, *C*_0_ is the detected C_2_H_2_ concentration in the inlet gas, and *V*_*m*_ is molar volume of gas.

The productivity calculation in breakthrough experiment is defined by:

4 $$p = \frac{{\mathop {\smallint }\nolimits_{t_1}^{t_2} V_e\left( t \right)dt}}{{V_m}} = \frac{{\mathop {\smallint }\nolimits_{t_1}^{t_2} \frac{{u_e(t) \times y_e(t) \times F}}{{u_i}}dt}}{{V_m}} = \frac{{F \times y_{{\mathrm{C}}_2{\mathrm{H}}_4} \times \mathop {\smallint }\nolimits_{t_1}^{t_2} \frac{{C(t)}}{{C_0}}dt}}{{V_m}}$$

where *p* is the C_2_H_4_ productivity in mmol/g, *t*_1_ is the C_2_H_4_ breakthrough time, *t*_2_ is the C_2_H_2_ breakthrough time for a specific C_2_H_2_ concentration, *u*_*e*_*(t)* is the transient linear velocity in outlet gas, *y*_*e*_*(t)* is the transient C_2_H_4_ volume fraction in outlet gas, *u*_*i*_ is the transient linear velocity in inlet gas, *F* is the inlet gas volume flow rate, $y_{{\mathrm{C}}_2{\mathrm{H}}_4}$ is the volume fraction of the C_2_H_4_ in the mixed gas, 0.99, $\mathop {\smallint }\nolimits_{t_1}^{t_2} \frac{{C(t)}}{{C_0}}dt$ is the integrated area between the C_2_H_4_ breakthrough curve and the *x* axis in range of *t*_1_ *t*o *t*_2_, *C(t)* is the detected C_2_H_4_ concentration in the outlet gas, *C*_0_ is the detected C_2_H_4_ concentration in the inlet gas, and *V*_*m*_ is molar volume of gas.

### Density-functional theory calculations

The static binding energy was calculated using the combination of first-principle density function theory (DFT) and plane-wave ultrasoft pseudopotential implemented in the Material Studio, CASTEP code^51^. A semi-empirical addition of dispersive forces to conventional DFT was included in the calculation to account for van der Waals interaction. Calculations were performed under the generalized gradient approximation (GGA) with Perdew–Burke–Ernzerhof (PBE) exchange correlation^28,30^. A cutoff energy of 544 eV while 2 × 2 × 1 k-point mesh for ZUL-200, 2 × 1 × 2 k-point mesh for ZUL-210, 2 × 2 × 1 k-point mesh for ZUL-220, and 2 × 1 × 2 k-point mesh for ZUL-100 all with smearing 0.2 eV were found to be enough for the total energy to converge within 1 × 10^−6^ eV atom ^−1^, and the calculation error are within 0.15 Å. The structure of all samples would be first optimized by the UFF force field implemented in the Materials Studio, Forcite module, using the experimentally-obtained single crystal structures as initial geometries and with a full structural relaxation that allows all atomic positions and unit cell parameters to vary. No remarkable difference was observed between the optimized structure and the experimental single crystal structure for ZUL-220, ZUL-200, and ZUL-100, and only a slight difference in the relative position of adjacent layers was observed for ZUL-210, suggesting a good match between the optimized structures with the experimentally determined ones. Then the isolated gas molecule was placed in the same cell dimensions as every sample crystal and was optimized and relaxed as references. Various guest gas molecules were finally introduced to different locations of the channel pore, followed by a full structural relaxation. More than eight initial configurations were optimized to ensure a more efficient energy landscape scanning for every MOF-guest complex, and the optimized configuration having the lowest energy was used as the global minimum for the subsequent analysis and calculation. The static binding energy (at *T* = 0 K) was then calculated: △*E* = *E*(MOF) + *E*(gas) − *E*(MOF + gas). We selected some benchmark materials including SIFSIX-1-Cu and SIFSIX-2-Cu-i to carry out the same DFT calculation as that for the ZUL materials, and the calculated structure and energy are in good agreement with those reported in the literature. For example, the acetylene binding energy of SIFSIX-1-Cu calculated by us is 47.2 kJ/mol, while the reported value in literature is 47.0 kJ/mol^28^. These results support that the comparison of binding energy in this work is feasible. When comparing the energy between hypothetical activated model and practical activated model, the unit cell of the models derived by practical activated materials was transformed to keep the number of atoms equal in both cases. Then the geometrical optimization of all crystal structures allows all atomic positions and unit cell parameters to vary. Only subtle changes in the framework atomic positions were observed, resulting in near-superimposable computed and experimental structures. The molecular surface electrostatic potential was calculated for the hydrogen-terminated fragment of the 2D nets in ZUL-220 and ZUL-200 which contains 546 atoms and 582 atoms (comprising 4*4 NbOF_5_^2−^ anions and 18 organic linkers), respectively, using Multiwfn 3.7 program^52,53^ and the GFN2-xTB method as implemented in the xtb code^54^, and the visualization was performed by VMD 1.9 program^55^.

### Fitting of pure component isotherms

The pure component isotherm data for C_2_H_2_ and C_2_H_4_ in ZUL-100 and ZUL-200 were fitted with the dual-site Langmuir-Freundlich (DSLF) equation.

5 $$q = q_{{\mathrm{A}},{\mathrm{sat}}}\frac{{b_{\mathrm{A}}p^{v_{\mathrm{A}}}}}{{1 + b_{\mathrm{A}}p^{v_{\mathrm{A}}}}} + q_{{\mathrm{B}},{\mathrm{sat}}}\frac{{b_{\mathrm{B}}p^{v_{\mathrm{B}}}}}{{1 + b_{\mathrm{B}}p^{v_{\mathrm{B}}}}}$$

Here, *q* is the gas uptake per mass of adsorbent (in mmol/g), *p* is the pressure of the bulk gas at equilibrium with the adsorption phase (in Pa), $q_{{\mathrm{A}},{\mathrm{sat}}}$ and $q_{{\mathrm{B}},{\mathrm{sat}}}$ are the saturation uptakes for site 1 and 2 (in mmol/g), $b_{\mathrm{A}}$and $b_{\mathrm{B}}$ are the affinity coefficients of site 1 and 2 (in Pa^−1^), and *v*_A_ and *v*_B_ are the deviations from an ideal homogeneous surface. The parameters that were obtained from fitting of the C_2_H_2_ and C_2_H_4_ adsorption isotherms are provided in Tables S9 and S10, respectively. All isotherms were fitted with *R*^2^ > 0.9999.

### Isosteric heat of adsorption

The experimental isosteric heat of adsorption (*Q*_st_) values for C_2_H_2_ and C_2_H_4_ in ZUL-100 and ZUL-200 were calculated using Virial-type expression:

6 $${\mathrm{ln}}P = {\mathrm{ln}}N + 1/T\mathop {\sum}\limits_{i = 0}^m {a_iN^i + \mathop {\sum }\limits_{i = o}^n \left( {\begin{array}{*{20}{c}} n \\ k \end{array}} \right)b_iN^i}$$

7 $$Q_{{\mathrm{st}}} = - R\mathop {\sum}\limits_{i = 0}^m {a_iN^i}$$

where *P* is the pressure described in mmHg, *N* is the adsorption capacity in mmol/g, *T* is the temperature in K, *a*_i_ and *b*_i_ are Virial coefficients, and *m* and *n* are the numbers of coefficients used to describe the isotherms. *Q*_st_ is the coverage-dependent enthalpy of adsorption, and *R* is the universal gas constant.

### IAST calculation of adsorption selectivity

The adsorption selectivity for C_2_H_2_/C_2_H_4_ separation is defined by^56^

8 $$S_{{\mathrm{ads}}} = \frac{{q_1/q_2}}{{p_1/p_2}}$$

where *q*_1_ and *q*_2_ are the molar loadings in the adsorbed phase in equilibrium with the bulk gas phase, *p*_1_ and *p*_2_ are partial pressure.

### Separation potential calculation of fixed bed adsorber

This separation potential, ▵*q*, represents the maximum number of moles of pure component 2 (the less strongly adsorbed species) that can be recovered in the gas phase per gram of adsorbent in the fixed bed. The separation potential of adsorbers in fixed bed for C_2_H_2_/C_2_H_4_ separation is defined by^47,48^

9 $$\Delta q = q_1\frac{{y_2}}{{y_1}} - q_2$$

where *q*_1_ and *q*_2_ are the molar loadings for mixture adsorption, calculated from the IAST in mmol/g, *y*_2_ and *y*_1_ are molar fractions in the binary mixture gas.

### Transient breakthrough simulations

Transient breakthrough simulations were carried out for binary 1/99 C_2_H_2_(1)/C_2_H_4_(2) mixtures at 298 K and 1 bar, using the methodology described in earlier publications^47,48,57,58^. For the breakthrough simulations, the following parameter values were used: length of packed bed, *L* = 0.3 m; voidage of packed bed, *ε* *=* 0.4, interstitial gas velocity at inlet, *ν* = 0.1 m/s; superficial gas velocity at inlet, *u* = 0.04 m/s. In the breakthrough simulations, the intra-crystalline diffusional influences are ignored. Also ignored in the transient breakthrough simulations are axial dispersion effects in the tube. The transient breakthrough simulation results are presented in terms of a dimensionless time, *τ*, defined by dividing the actual time, *t*, by the characteristic time, $\frac{{L\varepsilon }}{u}$. And the productivity of purified C_2_H_4_ expressed in moles (or Liter at STP) per gram of adsorbent (or L of adsorbent) is uniquely determined by the parameter,

10 $$\frac{{({\mathrm{Time}}\;{\mathrm{in}}\;{\mathrm{minutes}}) \times ({\mathrm{Flow}}\;{\mathrm{rate}}\;{\mathrm{L}}/\min \;{\mathrm{at}}\;{\mathrm{STP}})}}{{({\mathrm{g}}\;{\mathrm{MOF}}\;{\mathrm{packed}}\;{\mathrm{in}}\;{\mathrm{tube}})}} = {\mathrm{L}}/{\mathrm{g}}$$

11 $$\left( {{\mathrm{Flow}}\;{\mathrm{rate}}\;{\mathrm{L}}/\min \;{\mathrm{at}}\;{\mathrm{STP}}} \right) = ({\mathrm{Superficial}}\;{\mathrm{gas}}\;{\mathrm{velocity}}) \times ({\mathrm{Cross}}\;{\mathrm{sectional}}\;{\mathrm{area}}) \times \left( {\frac{{273}}{{298}}} \right)$$

12 $$\left( {{\mathrm{g}}\;{\mathrm{MOF}}\;{\mathrm{packed}}\;{\mathrm{in}}\;{\mathrm{tube}}} \right) = (1 - \varepsilon ) \times ({\mathrm{Length}}\;{\mathrm{of}}\;{\mathrm{packed}}\;{\mathrm{tube}})\\ \times ({\mathrm{Cross}}\;{\mathrm{sectional}}\;{\mathrm{area}}) \times ({\mathrm{Crystal}}\;{\mathrm{framework}}\;{\mathrm{density}})$$

### Configurational-Bias Monte Carlo (CBMC) simulations

Configurational-Bias Monte Carlo (CBMC) simulations were carried out to determine the adsorption isotherms for unary C_2_H_2_, unary C_2_H_4_, and 1/99 C_2_H_2_/C_2_H_4_ mixtures in ZUL-100 and ZUL-200 at 298 K. The simulation methodologies are the same as detailed in earlier publications^59–65^. The ZUL-100 and ZUL-200 structures were considered to be rigid in the simulations. The unit cell was constructed using the cif files obtained by the crystallography characterization. The simulation box for conducting CBMC simulations consisted of 3 × 2 × = 18 unit cells. The interactions between adsorbed molecules are described with Lennard–Jones terms. For the atoms in the host metal organic framework, the generic UFF^66^ and DREIDING^67^ force fields were used; the Lennard–Jones parameters $\sigma _{{\mathrm{host}}}$, $\frac{{\varepsilon _{{\mathrm{host}}}}}{{k_B}}$ values are specified in Table S14. The united atom model was used to describe –CH groups in C_2_H_2_, and –CH_2_ groups in C_2_H_4_. The Lennard–Jones parameters for the –CH groups in C_2_H_2_ were taken from Gautam et al.^68^ The Lennard–Jones parameters for the –CH_2_ groups in C_2_H_4_ were taken from Ban et al.^69^ The Lennard–Jones parameters $\sigma _{{\mathrm{guest}}}$, $\frac{{\varepsilon _{{\mathrm{guest}}}}}{{k_B}}$ are tabulated in Table S15.

The Lorentz–Berthelot mixing rules were applied for calculating the Lennard–Jones parameters describing guest-host interactions:

13 $$\sigma _{{\mathrm{guest}} - {\mathrm{host}}} = \frac{{\left( {\sigma _{{\mathrm{guest}}} + \sigma _{{\mathrm{host}}}} \right)}}{2}$$

14 $$\frac{{\varepsilon _{{\mathrm{guest}} - {\mathrm{host}}}}}{{k_B}} = \sqrt {\frac{{\varepsilon _{{\mathrm{guest}}}}}{{k_B}} \times \frac{{\varepsilon _{{\mathrm{host}}}}}{{k_B}}}$$

The success of the applicability of the Lorentz–Berthelot mixing rules for interaction of –CH_2_ group with framework atoms of zeolites has also been established by Ban et al.^69^ by comparison of the experimental data on adsorption of alkenes in a wide variety of zeolites (with different pore sizes) with corresponding CBMC simulations. Gautam et al.^68^ have applied the Lorentz–Berthelot mixing rules for calculating interaction of –CH group with framework atoms of NaY zeolite for performing MD simulations of C_2_H_2_ in NaY zeolite. It is important to stress that there have been no other published works to verify the applicability of the Lorentz–Berthelot mixing rule for –CH group with framework atoms of either zeolites or MOFs.

The interactions of the guest pseudo-atoms with the F atoms of the framework are dominant. For interactions of the –CH_2_ groups in C_2_H_4_ with the F atoms of the frameworks, the best fit value of $\frac{{\varepsilon _{{\mathrm{CH}}2 - F}}}{{k_B}}$ was nearly identical to the value determined from the above equation, i.e., $\frac{{\varepsilon _{{\mathrm{CH}}2 - F}}}{{k_B}} = \sqrt {92.50 \times 36.4872} = 58.09529$ K, which is in line with earlier work of Ban et al.^69^ that have verified the applicability of the Lorentz–Berthelot rule for estimating $\frac{{\varepsilon _{{\mathrm{CH}}2 - F}}}{{k_B}}$. In the case of the interaction of –CH groups in C_2_H_2_ with the F atoms of the frameworks, the value of $\frac{{\varepsilon _{{\mathrm{CH}} - F}}}{{k_B}}$ had to be adjusted, i.e., fitted, to a value of 275.727 K, which is 6 times the value determined from the Lorentz–Berthelot mixing rule (it should be noted that this scaling factor is artificial and cannot be used for any other simulations except “fitting” the experimental data to support the reliability of the IAST calculations in this work). The need for fitting of the interaction parameter $\frac{{\varepsilon _{{\mathrm{CH}} - F}}}{{k_B}}$ is a clear reflection of the fact that the Lorentz–Berthelot mixing rule has not been tested or verified in any earlier publications. Table S16 summarizes the values of the Lennard–Jones parameters for gues–F interactions that are used in the simulations. The Lennard–Jones potentials are shifted and cut at 12 Å. Since both ZUL-100 and ZUL-200 do not contain open metal sites, the electrostatic charge interactions are not considered.

Figure S37a presents CBMC simulation data (indicated by the red and green symbols) for the component loadings for adsorption of 1/99 C_2_H_2_/C_2_H_4_ mixtures in ZUL-100 at 298 K. The continuous solid black lines are IAST calculations of adsorption equilibrium using the dual-Langmuir fits of unary isotherms determined from CBMC. There is perfect agreement between CBMC mixture simulations and IAST calculations. Figure S37b presents CBMC simulated data (indicated by red symbols) on C_2_H_2_/C_2_H_4_ adsorption selectivities in ZUL-100. The CBMC selectivity data are compared with IAST calculations (indicated by the continuous solid lines) using unary isotherm data determined from CBMC (black solid lines) and also from experiments (green solid lines). The CBMC determined selectivities are in reasonably good agreement with experimentally determined selectivity.

Figure S38a presents CBMC simulation data (indicated by the red and green symbols) for the component loadings for adsorption of 1/99 C_2_H_2_/C_2_H_4_ mixtures in ZUL-200 at 298 K. The continuous solid black lines are IAST calculations of adsorption equilibrium using the dual-Langmuir fits of unary isotherms determined from CBMC. There is perfect agreement between CBMC mixture simulations and IAST calculations. Figure S38b presents CBMC simulated data (indicated by red symbols) on C_2_H_2_/C_2_H_4_ adsorption selectivities in ZUL-200. The CBMC selectivity data are compared with IAST calculations (indicated by the continuous solid lines) using unary isotherm data from CBMC (black solid lines) and also from experiments (green solid lines). The CBMC determined selectivities are in reasonably good agreement with experimentally determined selectivity.

## Supplementary information

Supplementary Information

## Data Availability

Crystallographic data for ZUL-100, ZUL-200, activated ZUL-200, ZUL-210, activated ZUL-210, ZUL-220, activated ZUL-220, and C_2_H_2_@ZUL-200 are available free of charge from the Cambridge Crystallographic Data Centre via www.ccdc.cam.ac.uk/data_request/cif, under reference numbers CCDC 1974868 to 1974875. The data shown in the plots and that support the findings of this study are available from the corresponding author on reasonable request.

## References

[1] International Energy Agency. Energy Technology Perspectives 2017. http://www.iea.org/etp2017 (2017).

[2] Kitagawa S (2015). Porous materials and the age of gas. Angew. Chem. Int. Ed..

[3] Sholl DS, Lively RP (2016). Seven chemical separations to change the world. Nature.

[4] Magdalena ML (2012). Understanding carbon dioxide adsorption on univalent cation forms of the flexible zeolite Rho at conditions relevant to carbon capture from flue gases. J. Am. Chem. Soc..

[5] Datta SJ (2015). CO_2_ capture from humid flue gases and humid atmosphere using a microporous copper silicate. Science.

[6] Hao G, Li W, Qian D, Lu A (2010). Rapid synthesis of nitrogen-doped porous carbon monolith for CO_2_ capture. Adv. Mater..

[7] Furukawa H, Cordova KE, O’Keeffe M, Yaghi OM (2013). The chemistry and applications of metal-organic frameworks. Science.

[8] Bao Z (2018). Molecular sieving of ethane from ethylene through the molecular cross-section size differentiation in gallate-based metal-organic frameworks. Angew. Chem. Int. Ed..

[9] Bloch ED (2012). Hydrocarbon separations in a metal-organic framework with open Iron(II) coordination sites. Science.

[10] Das MC (2012). Interplay of metalloligand and organic ligand to tune micropores within isostructural mixed-metal organic frameworks (M’MOFs) for their highly selective separation of chiral and achiral small molecules. J. Am. Chem. Soc..

[11] Chen K (2016). Benchmark C_2_H_2_/CO_2_ and CO_2_/C_2_H_2_ separation by two closely related hybrid ultramicroporous materials. Chem.

[12] Liao P, Huang N, Zhang W, Zhang J, Chen X (2017). Controlling guest conformation for efficient purification of butadiene. Science.

[13] Xiang S (2010). Open metal sites within isostructural metal-organic frameworks for differential recognition of acetylene and extraordinarily high acetylene storage capacity at room temperature. Angew. Chem. Int. Ed..

[14] Li J (2019). Metal-organic framework containing planar metal-binding sites: efficiently and cost-effectively enhancing the kinetic separation of C_2_H_2_/C_2_H_4_. J. Am. Chem. Soc..

[15] Zhang Z (2017). Sorting of C4 olefins with interpenetrated hybrid ultramicroporous materials by combining molecular recognition and size-sieving. Angew. Chem. Int. Ed..

[16] Li L (2019). A robust squarate-based metal-organic framework demonstrates record-high affinity and selectivity for xenon over krypton. J. Am. Chem. Soc..

[17] Wang S (2019). Highly selective, high capacity separation of *o*-xylene from C8 aromatics by a switching adsorbent layered material. Angew. Chem. Int. Ed..

[18] Tchalala MR (2019). Fluorinated MOF platform for selective removal and sensing of SO_2_ from flue gas and air. Nat. Commun..

[19] Zhou D (2019). Intermediate-sized molecular sieving of styrene from larger and smaller analogues. Nat. Mater..

[20] Lin R (2018). Molecular sieving of ethylene from ethane using a rigid metal-organic framework. Nat. Mater..

[21] Hao H (2018). Simultaneously trapping C_2_H_2_ and C_2_H_6_ into a robust metal-organic framework from a ternary mixture of C_2_H_2_/C_2_H_4_/C_2_H_6_ for purification of C_2_H_4_. Angew. Chem. Int. Ed..

[22] Cadiau A, Adil K, Bhatt PM, Belmabkhout Y, Eddaoudi M (2016). A metal-organic framework−based splitter for separating propylene from propane. Science.

[23] Li L (2018). Ethane/ethylene separation in a metal-organic framework with iron-peroxo sites. Science.

[24] Liang W (2019). A tailor-made interpenetrated MOF with exceptional carbon-capture performance from flue gas. Chem.

[25] Yang L (2018). An asymmetric anion-pillared metal-organic framework as a multisite adsorbent enables simultaneous removal of propyne and propadiene from propylene. Angew. Chem. Int. Ed..

[26] Peng Y (2018). Robust ultramicroporous metal-organic frameworks with benchmark affinity for acetylene. Angew. Chem. Int. Ed..

[27] Bajpai A (2017). The effect of centred versus offset interpenetration on C_2_H_2_ sorption in hybrid ultramicroporous materials. Chem. Comm..

[28] Cui X (2016). Pore chemistry and size control in hybrid porous materials for acetylene capture from ethylene. Science.

[29] Zhang Z (2018). Hexafluorogermanate (GeFSIX) anion-functionalized hybrid ultramicroporous materials for efficiently trapping acetylene from ethylene. Ind. Eng. Chem. Res..

[30] Li B (2017). An ideal molecular sieve for acetylene removal from ethylene with record selectivity and productivity. Adv. Mater..

[31] O’Nolan D, Kumar A, Zaworotko MJ (2017). Water vapor sorption in hybrid pillared square grid materials. J. Am. Chem. Soc..

[32] O’Nolan D (2018). Impact of partial interpenetration in a hybrid ultramicroporous material on C_2_H_2_/C_2_H_4_ separation performance. Chem. Commun..

[33] Sato R (2014). Self-accelerating CO sorption in a soft nanoporous crystal. Science.

[34] Mohideen M (2011). Protecting group and switchable pore-discriminating adsorption properties of a hydrophilic-hydrophobic metal-organic framework. Nat. Chem..

[35] McHugh L (2018). Hydrolytic stability in hemilabile metal-organic frameworks. Nat. Chem..

[36] Tanaka D (2010). Rapid preparation of flexible porous coordination polymer nanocrystals with accelerated guest adsorption kinetics. Nat. Chem..

[37] Hijikata Y (2011). Differences of crystal structure and dynamics between a soft porous nanocrystal and a bulk crystal. Chem. Commun..

[38] Sakaida S (2016). Crystalline coordination framework endowed with dynamic gate-opening behavior by being downsized to a thin film. Nat. Chem..

[39] Stylianou K (2012). Dimensionality transformation through paddlewheel reconfiguration in a flexible and porous Zn-based metal-organic frameworks. J. Am. Chem. Soc..

[40] Gurunatha K, Maji T (2009). Guest-induced irreversible sliding in a flexible 2D rectangular grid with selective sorption characteristics. Inorg. Chem..

[41] Cheng Y (2011). Tuning of gate opening of an elastic layered structure MOF in CO_2_ sorption with a trace of alcohol molecules. Langmuir.

[42] Sanii R, Hua C, Patyk E, Zaworotko MJ (2019). Solvent-directed control over the topology of entanglement in square lattice (sql) coordination networks. Chem. Commun..

[43] Lin R (2017). Optimized separation of acetylene from carbon dioxide and ethylene in a microporous material. J. Am. Chem. Soc..

[44] Kondo A (2006). Novel expansion shrinkage modulation of 2D layered MOF triggered by clathrate formation with CO_2_ molecules. Nano Lett..

[45] Brigatti, M. & Mottana, A. *Layered Mineral Structures and Their Application in Advanced Technologies* (Mineralogical Society of Great Britain & Ireland, London, 2011).

[46] Elsaidi S, Mohamed M, Banerjee D, Thallapally P (2018). Flexibility in metal-organic frameworks: a fundamental understanding. Coord. Chem. Rev..

[47] Krishna R (2017). Screening metal-organic frameworks for mixture separations in fixed-bed adsorbers using a combined selectivity/capacity metric. RSC Adv..

[48] Krishna R (2018). Methodologies for screening and selection of crystalline microporous materials in mixture separations. Sep. Purif. Technol..

[49] Standardization Administration of the People’s Republic of China. *GB/T 7715-2003: **Ethylene for Industrial Use-Specification* (Standards Press of China, Beijing, 2003).

[50] ASTM International. *ASTM D5234-92(2017):* *Standard* Guide for Analysis of Ethylene Product (ASTM International, West Conshohocken, PA, 2017).

[51] Segall MD (2002). First-principles simulation: ideas, illustrations and the CASTEP code. J. Phys. Condens. Matter.

[52] Lu T, Chen F (2012). Multiwfn: a multifunctional wavefunction analyzer. J. Comput. Chem..

[53] Lu T, Chen F (2012). Quantitative analysis of molecular surface based on improved Marching Tetrahedra algorithm. J. Mol. Graph. Model..

[54] Bannwarth C, Ehlert S, Grimme S (2019). GFN2-xTB—an accurate and broadly parametrized self-consistent tight-binding quantum chemical method with multipole electrostatics and density-dependent dispersion contributions. J. Chem. Theory Comput..

[55] Humphrey W, Dalke A, Schulten K (1996). VMD-Visual molecular dynamics. J. Molec. Graph..

[56] Myers A, Prausnitz J (1965). Thermodynamics of mixed-gas adsorption. AIChE. J..

[57] Krishna R (2014). The Maxwell-Stefan description of mixture diffusion in nanoporous crystalline materials. Microporous Mesoporous Mater..

[58] Krishna R (2015). Methodologies for evaluation of metal-organic frameworks in separation applications. RSC Adv..

[59] Krishna R, van Baten JM (2011). In silico screening of metal-organic frameworks in separation applications. Phys. Chem. Chem. Phys..

[60] Krishna R, van Baten JM (2010). In silico screening of zeolite membranes for CO_2_ capture. J. Membr. Sci..

[61] Krishna R, van Baten JM (2010). Describing mixture diffusion in microporous materials under conditions of pore saturation. J. Phys. Chem. C..

[62] Krishna R, van Baten JM (2005). Diffusion of alkane mixtures in zeolites: Validating the Maxwell-Stefan formulation using MD simulations. J. Phys. Chem. B.

[63] Krishna R, van Baten JM (2008). Insights into diffusion of gases in zeolites gained from molecular dynamics simulations. Microporous Mesoporous Mater..

[64] Krishna R (2009). Describing the diffusion of guest molecules inside porous structures. J. Phys. Chem. C..

[65] Krishna R (2012). Diffusion in porous crystalline materials. Chem. Soc. Rev..

[66] Rappé AK (1992). UFF, a full periodic table force field for molecular mechanics and molecular dynamics simulations. J. Am. Chem. Soc..

[67] Mayo SL, Olafson BD, Goddard WA (1990). DREIDING: a generic force field for molecular simulations. J. Phys. Chem..

[68] Gautam S (2006). Diffusion of acetylene inside Na-Y zeolite: molecular dynamics simulation studies. Phys. Rev. E.

[69] Ban S (2007). Adsorption selectivity of benzene and propene mixtures for various zeolites. J. Phys. Chem. C..

