# Supplementary material for: Simultaneous interlayer and intralayer space control in two-dimensional metal−organic frameworks for acetylene/ethylene separation
Source: Nat Commun. 2020 Dec 7;11:6259. doi: 10.1038/s41467-020-20101-7 (PMC7721749; doi:10.1038/s41467-020-20101-7)
Supplement: Supplementary file 1 — Supplementary Information [file 41467_2020_20101_MOESM1_ESM.pdf]

Supplementary Information

Simultaneous interlayer and intralayer space control in two-dimensional metal−organic frameworks for acetylene/ethylene separation

Jin Shen[+,1], Xin He[+,2], Tian Ke[1], Rajamani Krishna[3], Jasper M. van Baten[3], Rundao Chen[1], Zongbi Bao[1], Huabin Xing[1], Mircea Dincă[2], Zhiguo Zhang[1], Qiwei Yang*[,1], and Qilong Ren[1]

[1]Key Laboratory of Biomass Chemical Engineering of Ministry of Education, College of Chemical and Biological Engineering, Zhejiang University, Hangzhou 310027, China. [2]Department of Chemistry, Massachusetts Institute of Technology, 77 Massachusetts Avenue, Cambridge, Massachusetts 02139, United States. [3]Van 't Hoff Institute for Molecular Sciences, University of Amsterdam, Science Park 904,1098 XH Amsterdam, The Netherlands.

[+]These authors contributed equally to this work.

e-mail: yangqw@zju.edu.cn

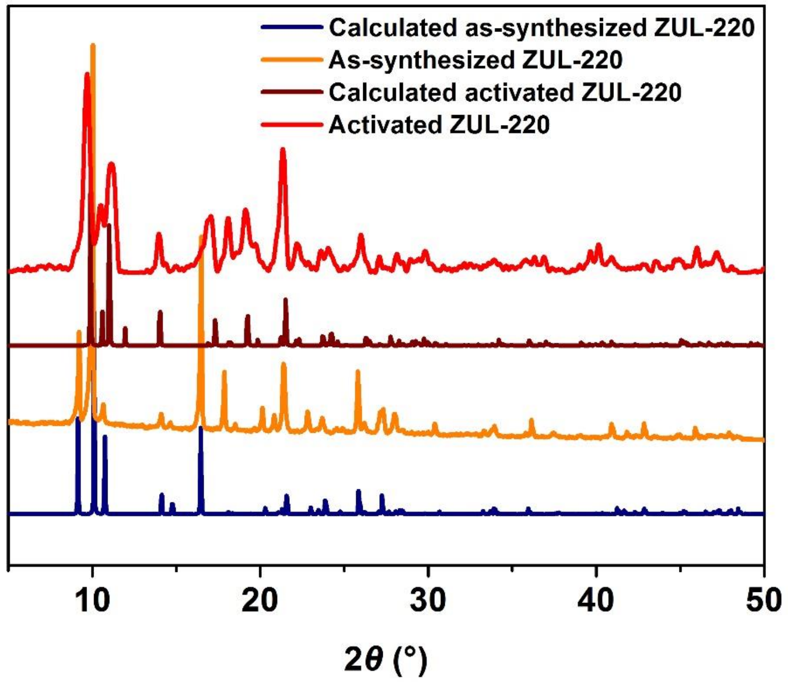

Supplementary Figure 1. The powder X-ray diffraction patterns of ZUL-220.

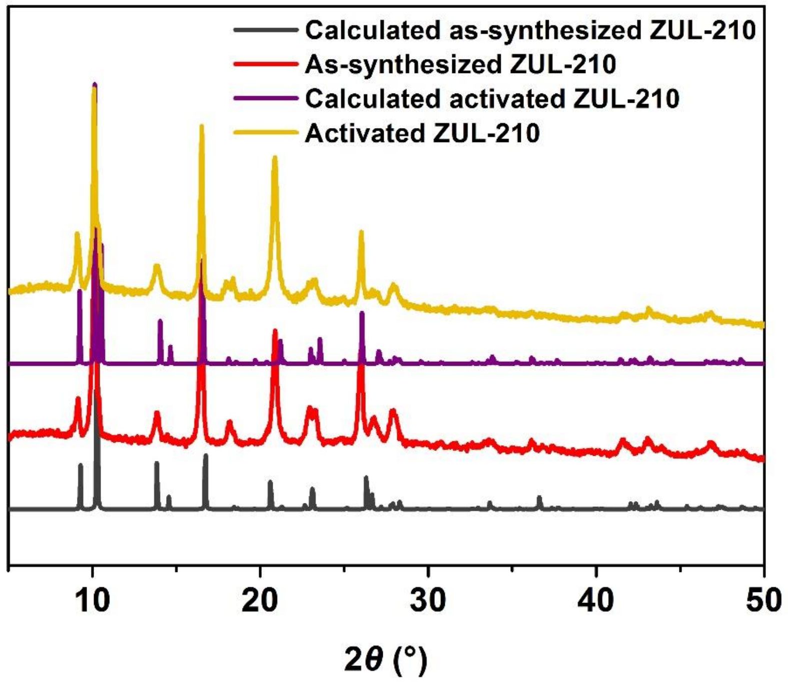

Supplementary Figure 2. The powder X-ray diffraction patterns of ZUL-210.

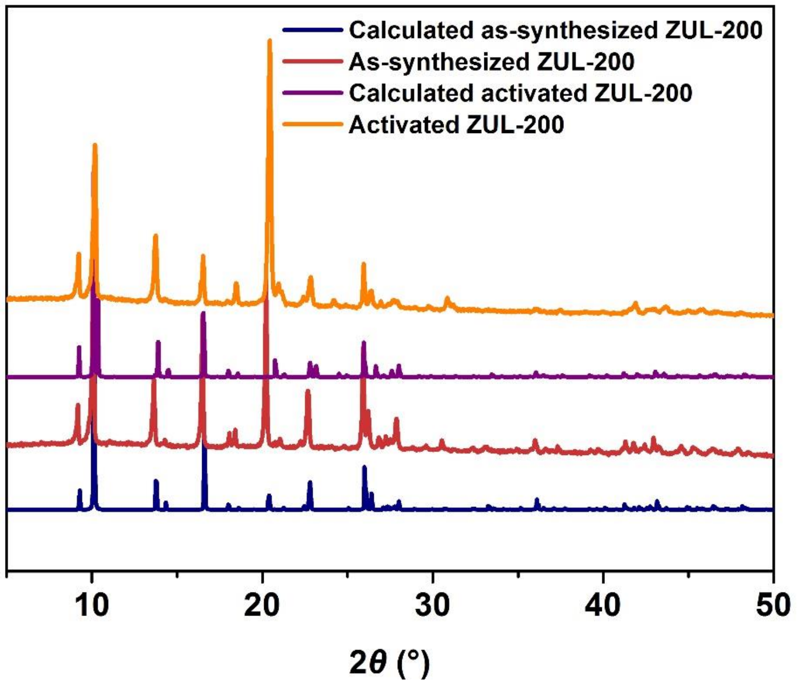

Supplementary Figure 3. The powder X-ray diffraction patterns of ZUL-200.

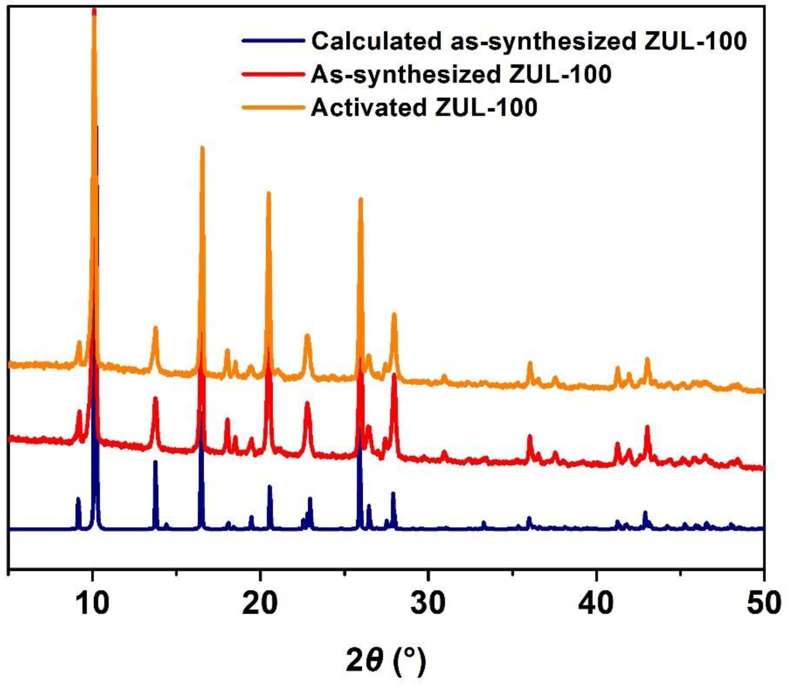

Supplementary Figure 4. The powder X-ray diffraction patterns of ZUL-100.

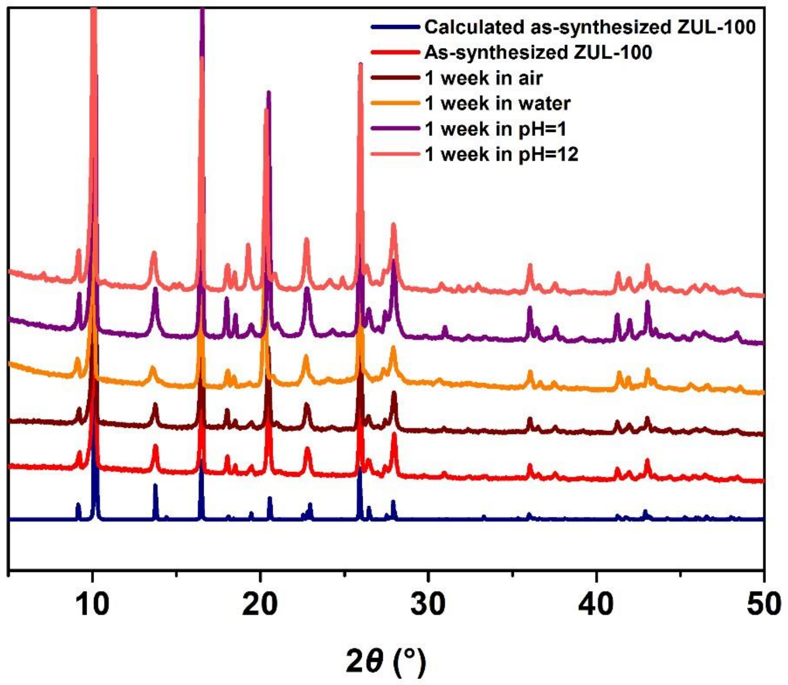

Supplementary Figure 5. The powder X-ray diffraction patterns of ZUL-100 after exposure to air, water, pH=1 aqueous solution and pH=12 aqueous solution.

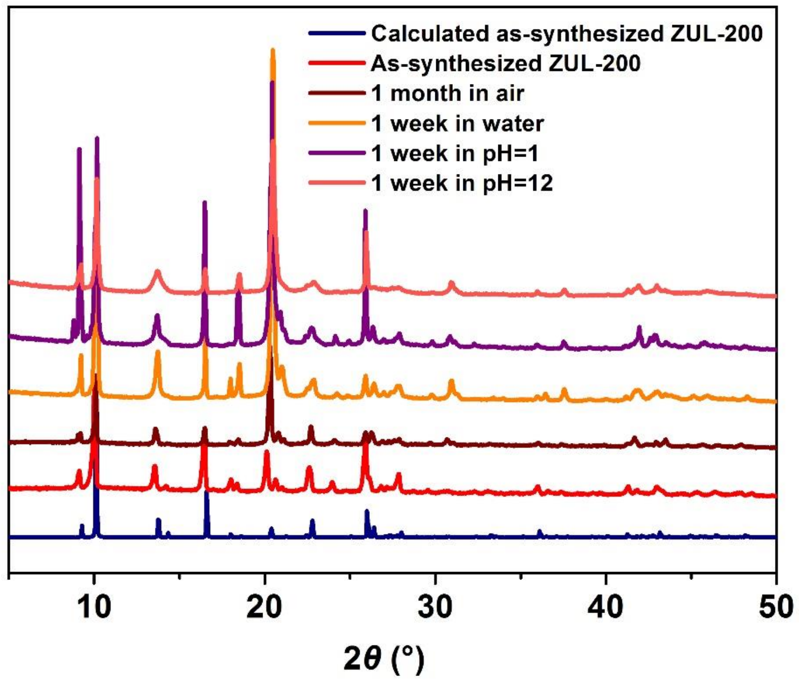

Supplementary Figure 6. The powder X-ray diffraction patterns of ZUL-200 after exposure to air, water, pH=1 aqueous solution and pH=12 aqueous solution.

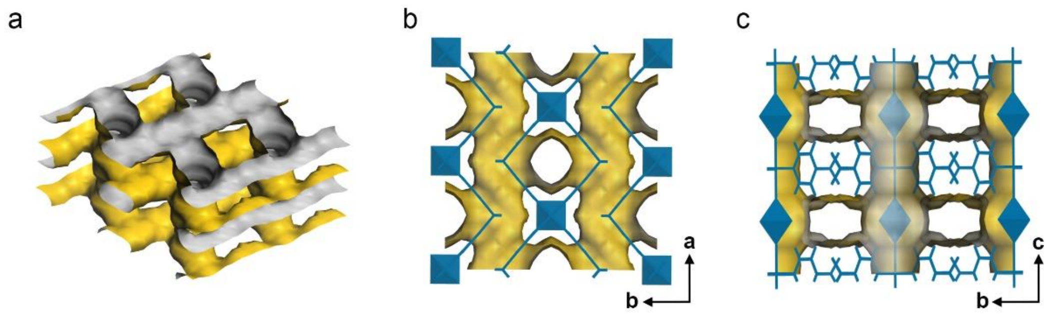

Supplementary Figure 7. Channel geometry of as-synthesized ZUL series materials (based on ZUL-100). Diagram of the cross-channel (a) viewed from (b) ***c*** axis and (c) ***a*** axis.

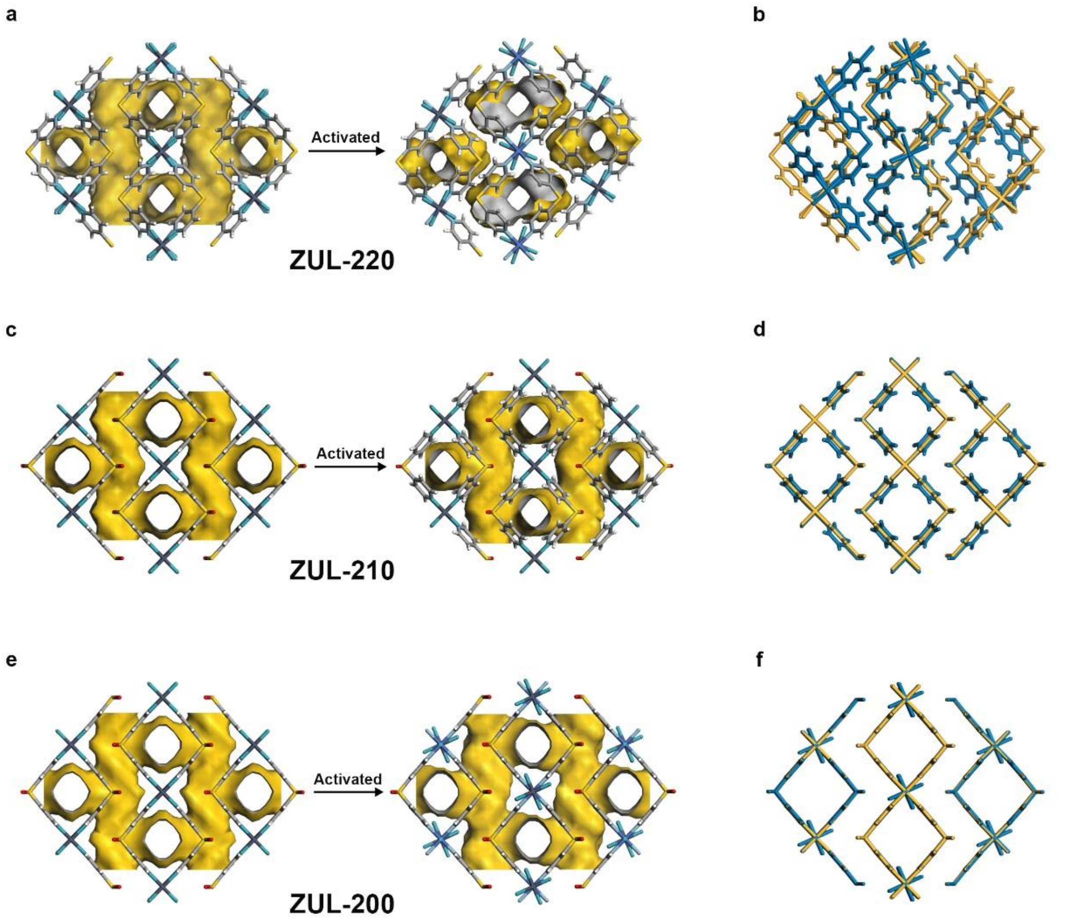

Supplementary Figure 8. (a) (c) (e) Porosity change during transformation from as-synthesized ZUL-220, ZUL-210, ZUL-200 to activated one, and (b) (d) (f) their corresponding cage units (yellow represents as-synthesized one and indigo represents activated one).

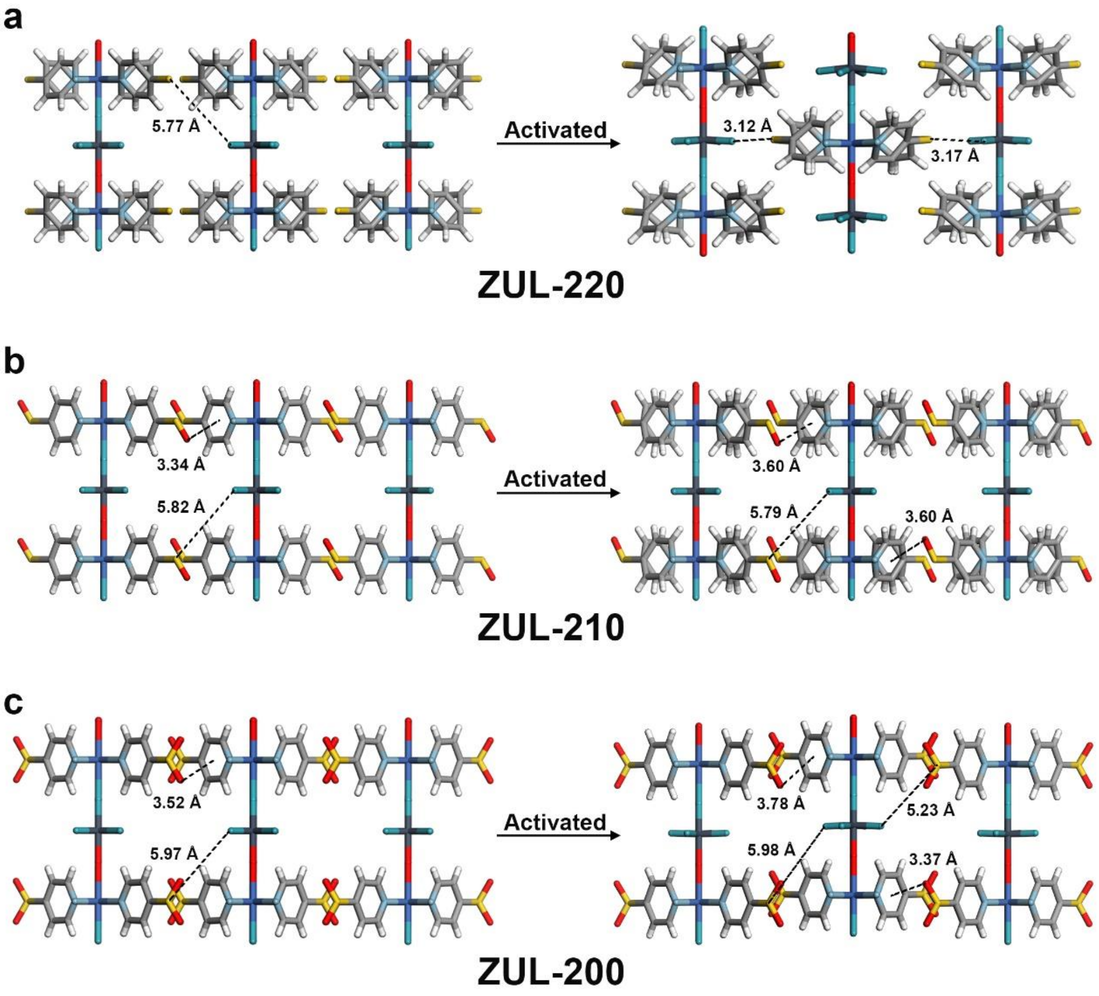

Supplementary Figure 9. The distances between S-F and O-pyridine ring change from as-synthesized to activated in (a) ZUL-220; (b) ZUL-210 and (c) ZUL-200.

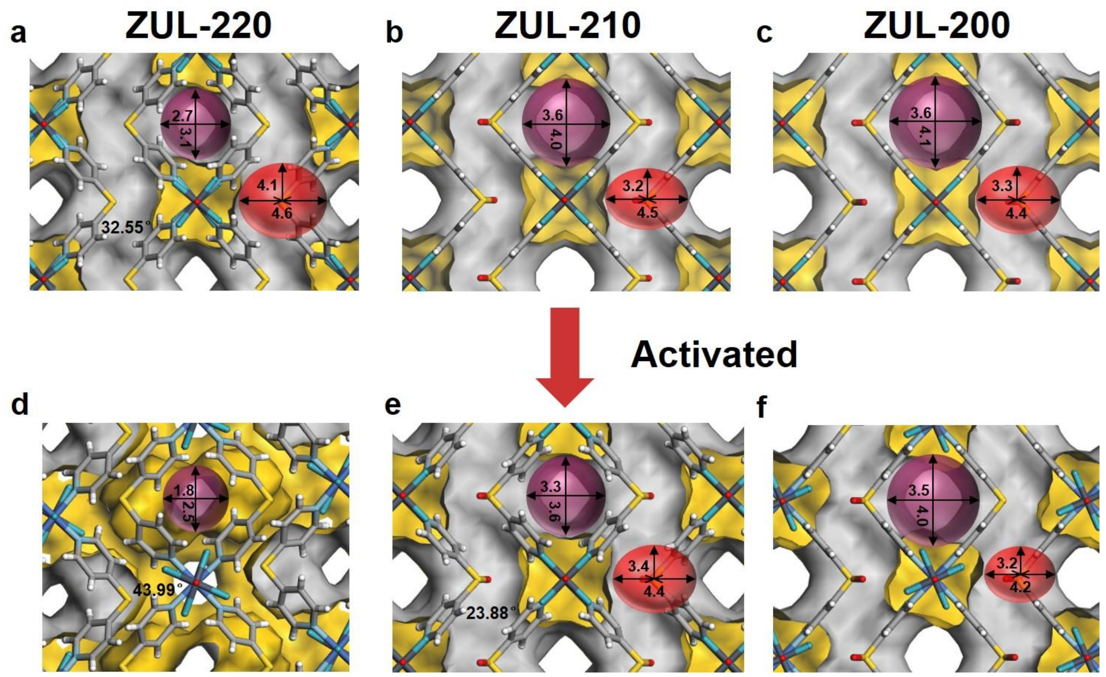

Supplementary Figure 10. Porosity change during transformation from as-synthesized ZUL-200, ZUL-210 and ZUL-200 to activated one.

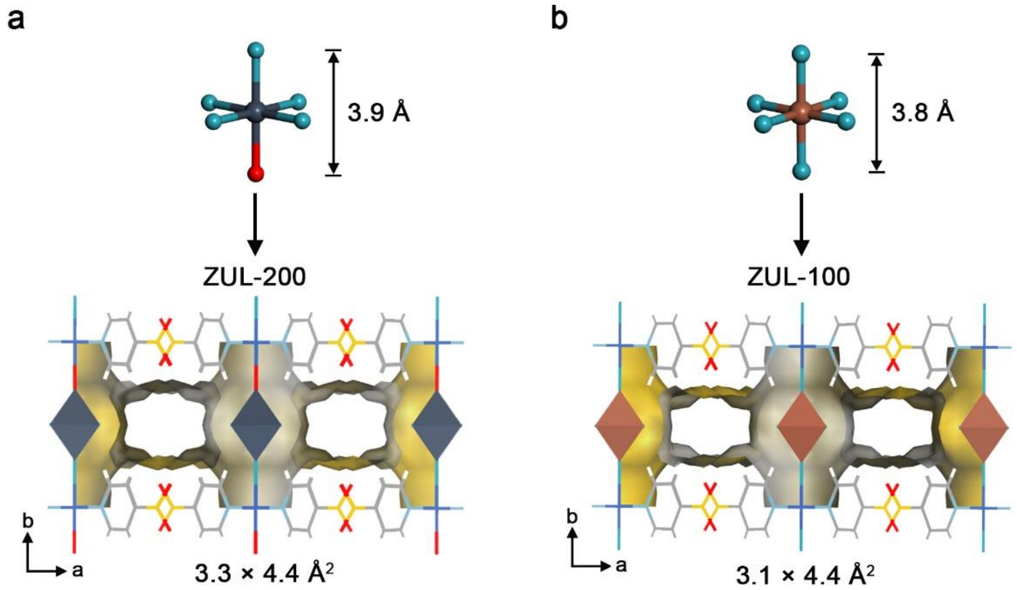

Supplementary Figure 11. The interlayer channels in ZUL-200 (a) and ZUL-100 (b).

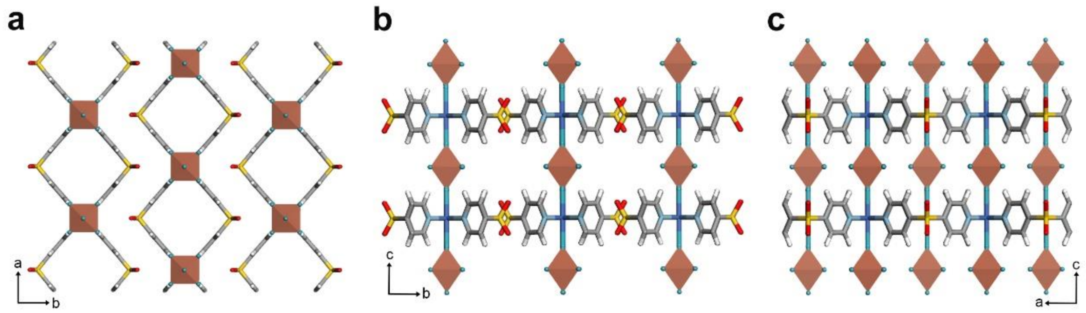

Supplementary Figure 12. The structure of ZUL-100 viewed from (a) ***c*** axis (b) ***a*** axis (c) ***b*** axis.

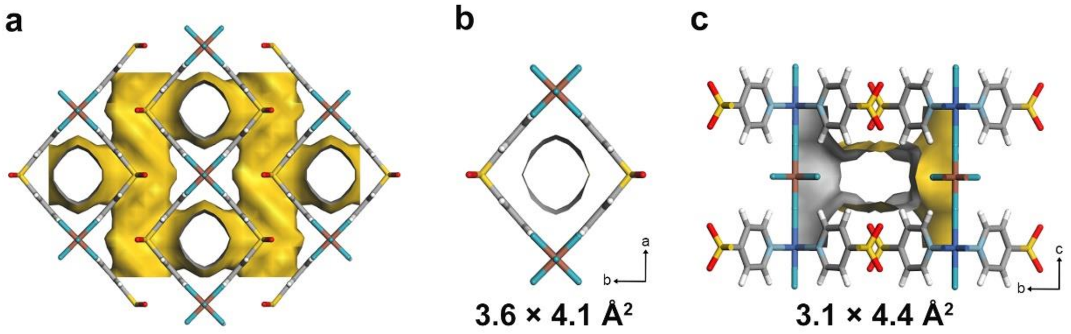

Supplementary Figure 13. Pore geometry of ZUL-100 (a) with intralayer (b) and interlayer (c) pore size.

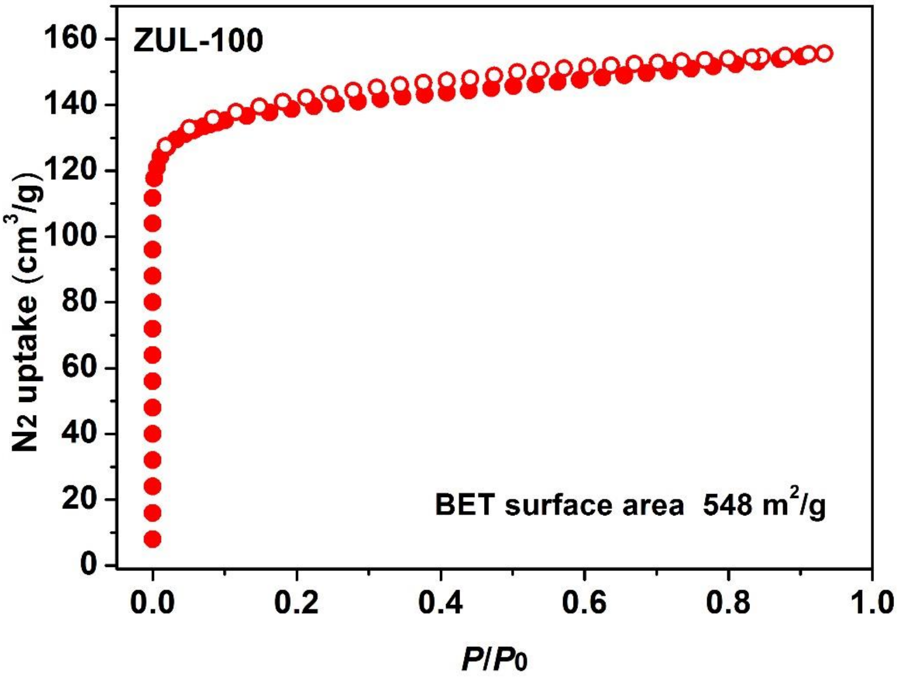

Supplementary Figure 14. N_2 adsorption isotherms of ZUL-100 at 77 K.

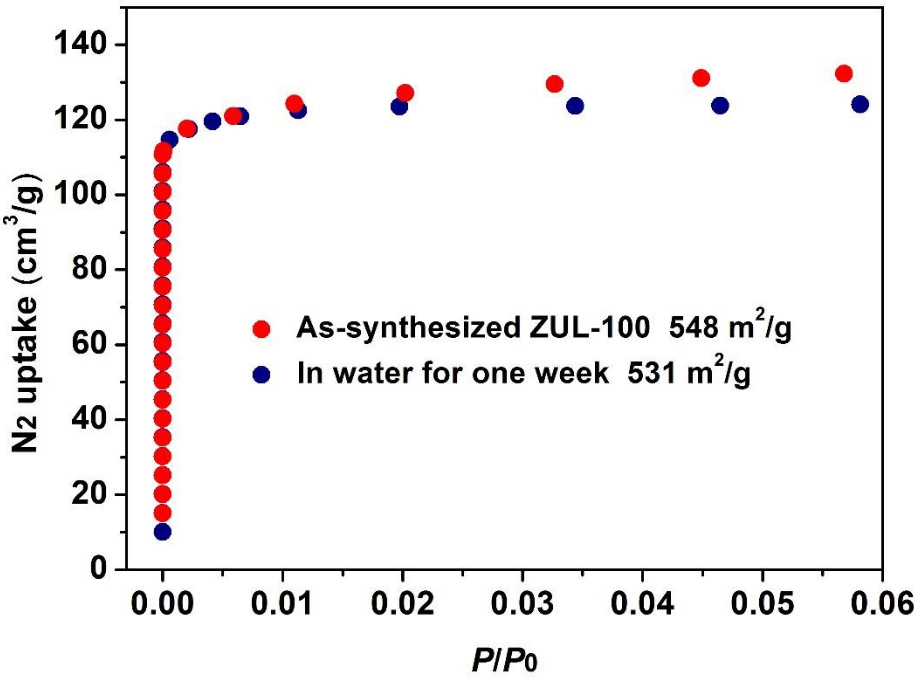

Supplementary Figure 15. 77 K N_2 adsorption isotherms of ZUL-100 (red) and ZUL-100 after soaking in water for one week.

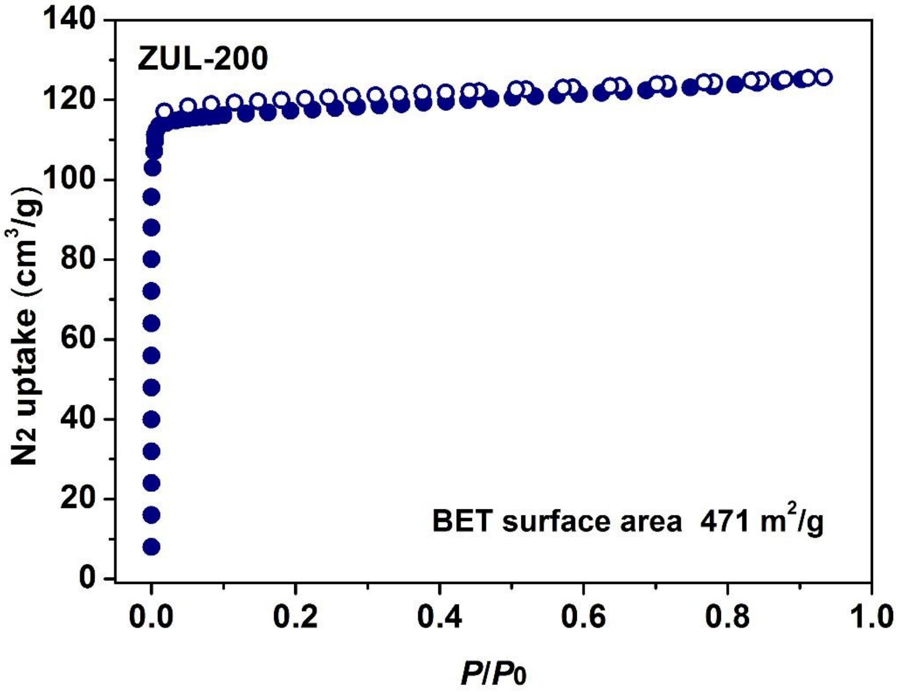

Supplementary Figure 16. N_2 adsorption isotherms of ZUL-200 at 77 K.

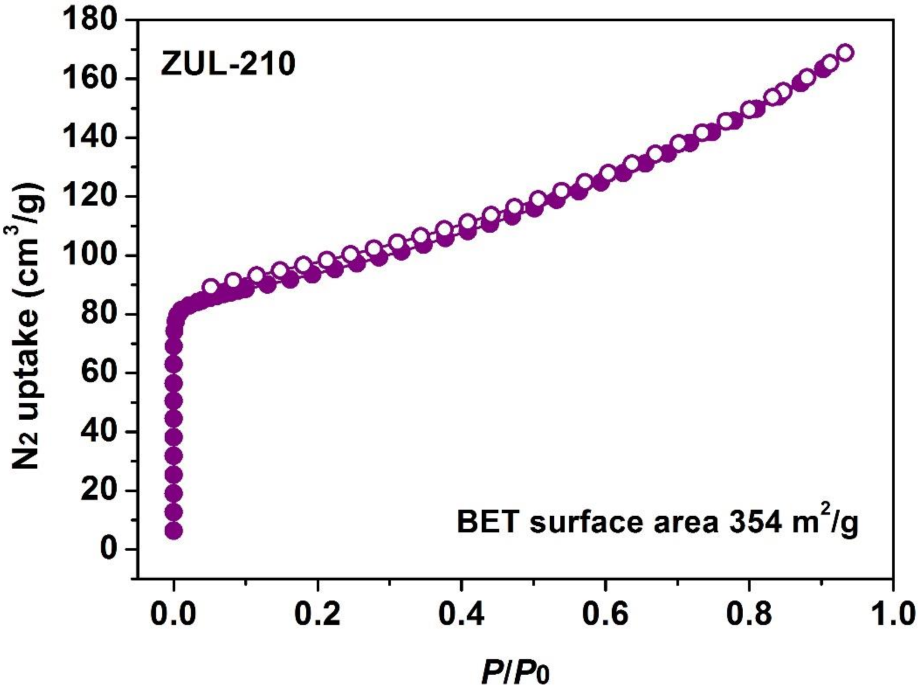

Supplementary Figure 17. N_2 adsorption isotherms of ZUL-210 at 77 K.

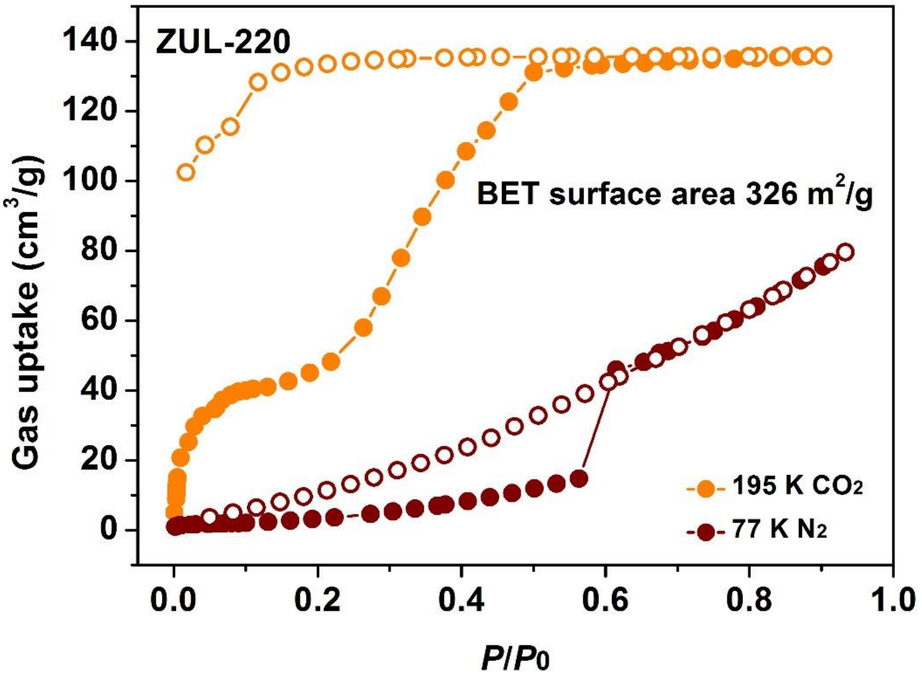

Supplementary Figure 18. 77 K N_2 adsorption isotherms and 196 K CO_2 adsorption isotherms of ZUL-220.

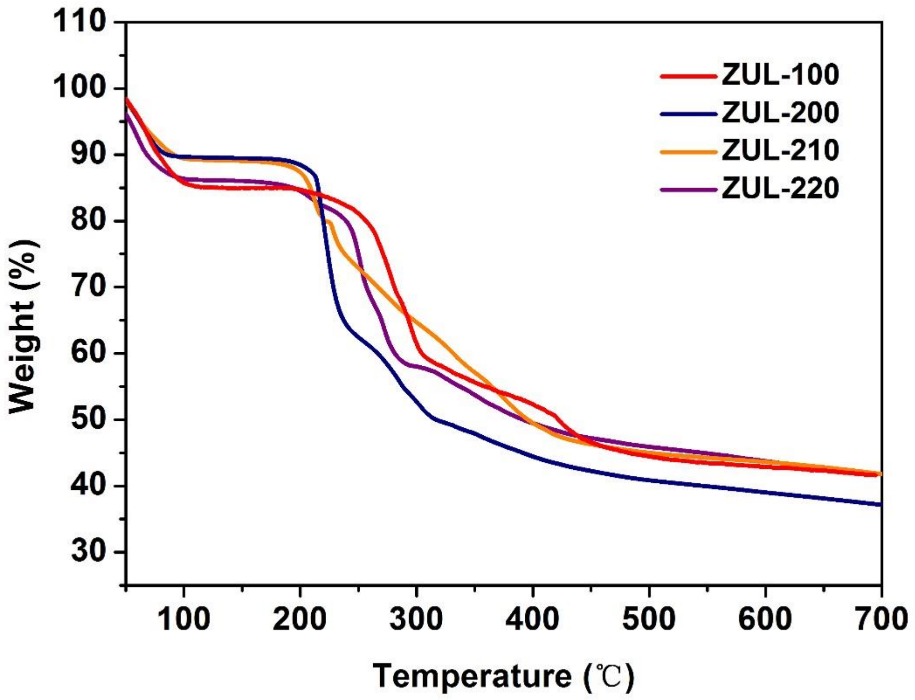

Supplementary Figure 19. TGA curves of as-synthesized ZUL-100, ZUL-200, ZUL-210 and ZUL-220.

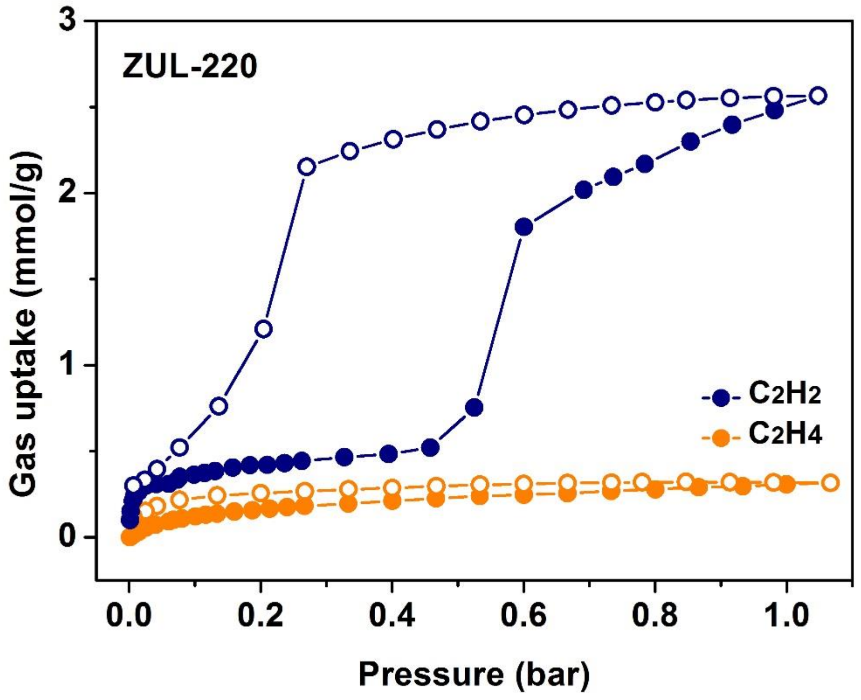

Supplementary Figure 20. Adsorption isotherms of C_2H_2 and C_2H_4 on ZUL-220 at 298 K. Adsorption and desorption are represented by closed and open symbols, respectively.

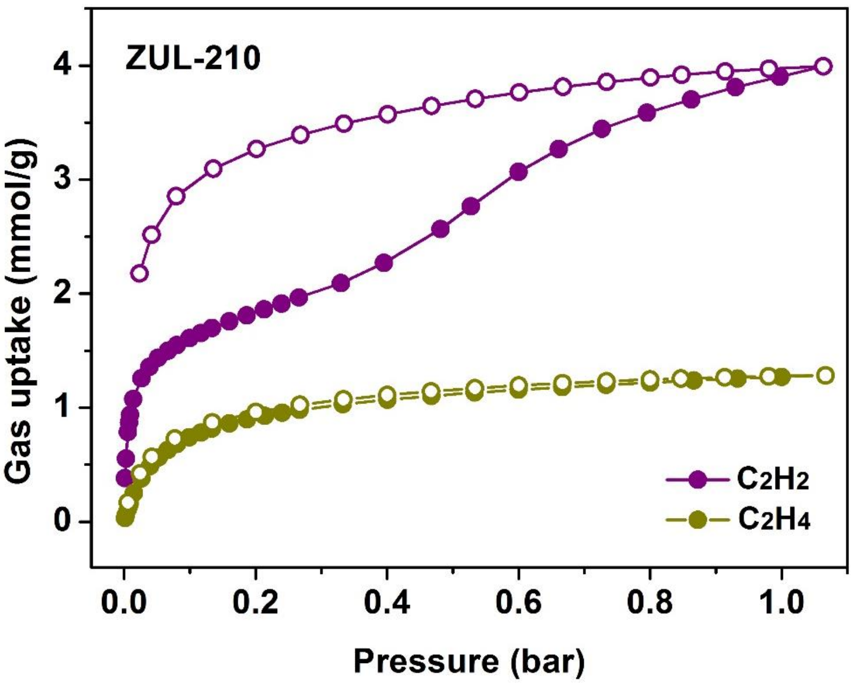

Supplementary Figure 21. The adsorption isotherms of C_2H_2 and C_2H_4 on ZUL-210 at 298 K. Adsorption and desorption are represented by closed and open symbols, respectively.

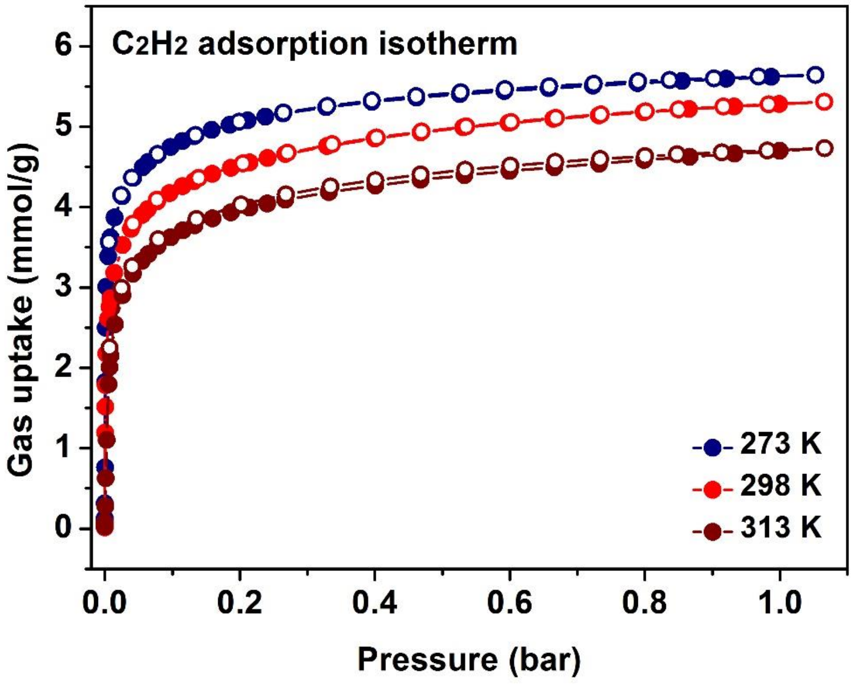

Supplementary Figure 22. The adsorption isotherms of C_2H_2 on ZUL-100 at temperature from 273 to 313 K. Adsorption and desorption are represented by closed and open symbols, respectively.

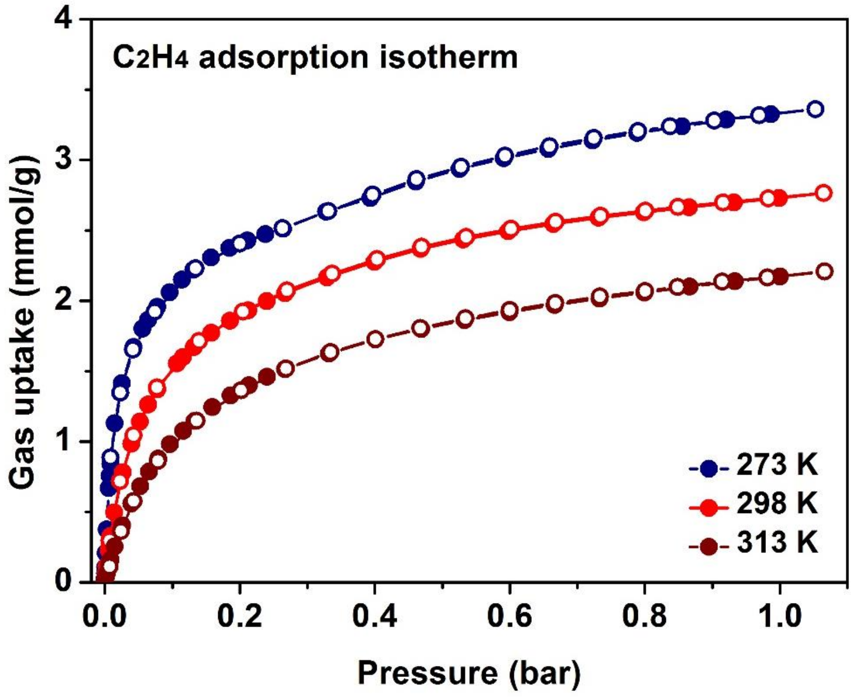

Supplementary Figure 23. The adsorption isotherms of C_2H_4 on ZUL-100 at temperature from 273 to 313 K. Adsorption and desorption are represented by closed and open symbols, respectively.

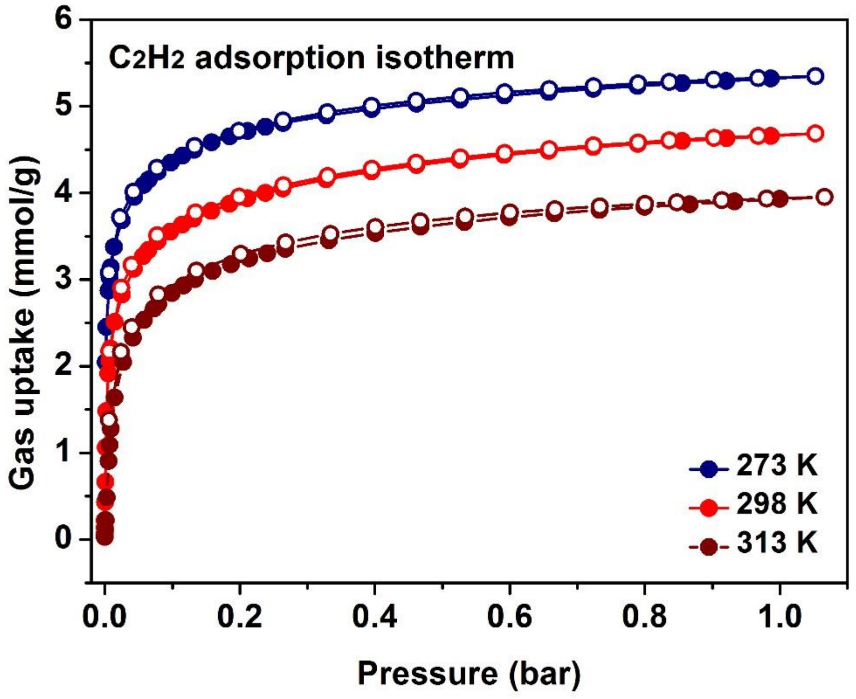

Supplementary Figure 24. The adsorption isotherms of C_2H_2 on ZUL-200 at temperature from 273 to 313 K. Adsorption and desorption are represented by closed and open symbols, respectively.

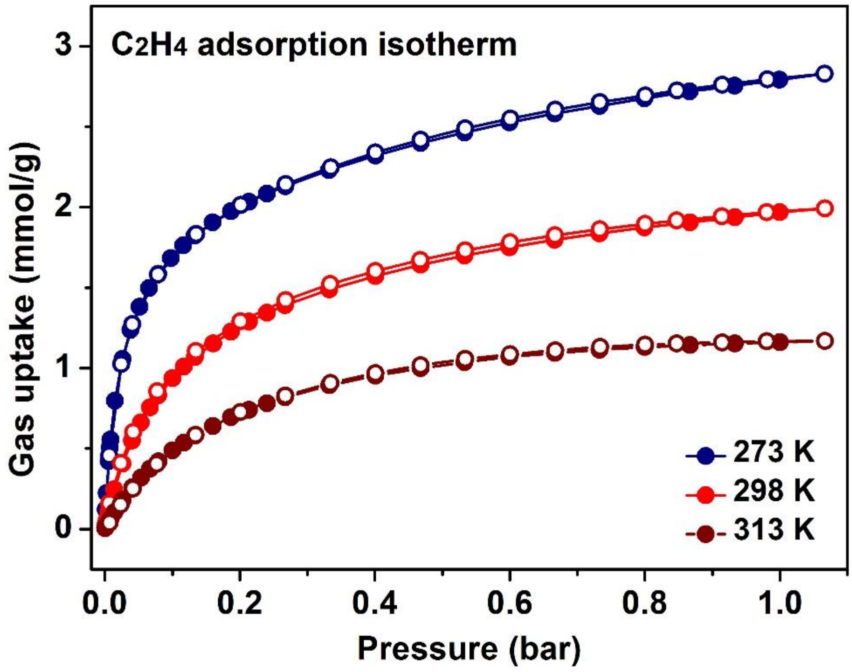

Supplementary Figure 25. The adsorption isotherms of C_2H_4 on ZUL-200 at temperature from 273 to 313 K. Adsorption and desorption are represented by closed and open symbols, respectively.

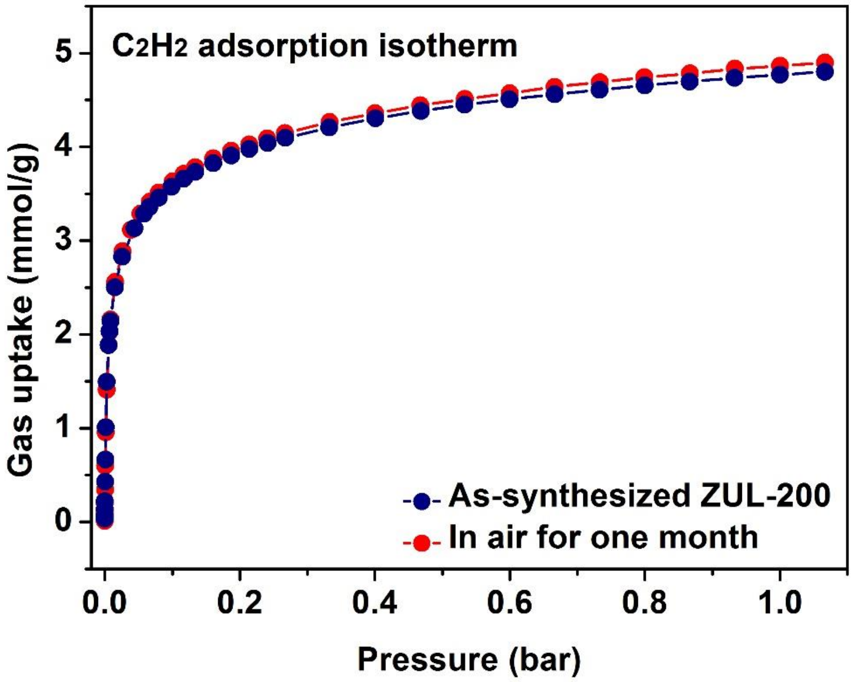

Supplementary Figure 26. The C_2H_2 adsorption isotherms on ZUL-200 after exposure to air for one month at 298 K. Adsorption and desorption are represented by closed and open symbols, respectively.

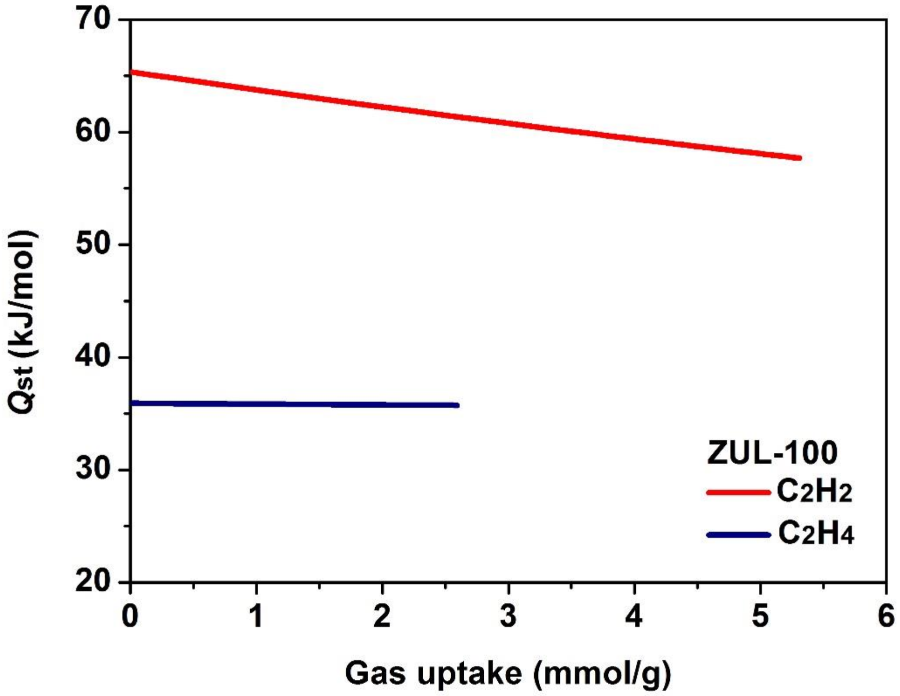

Supplementary Figure 27. Q_{st} of C_2H_2 and C_2H_4 adsorption in ZUL-100.

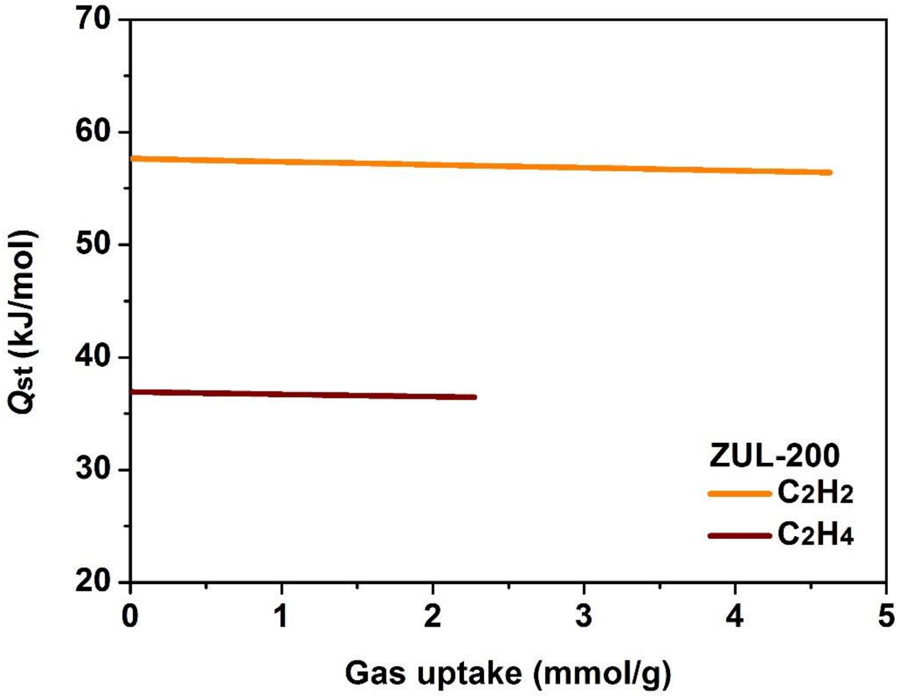

Supplementary Figure 28. Q_{st} of C_2H_2 and C_2H_4 adsorption in ZUL-200.

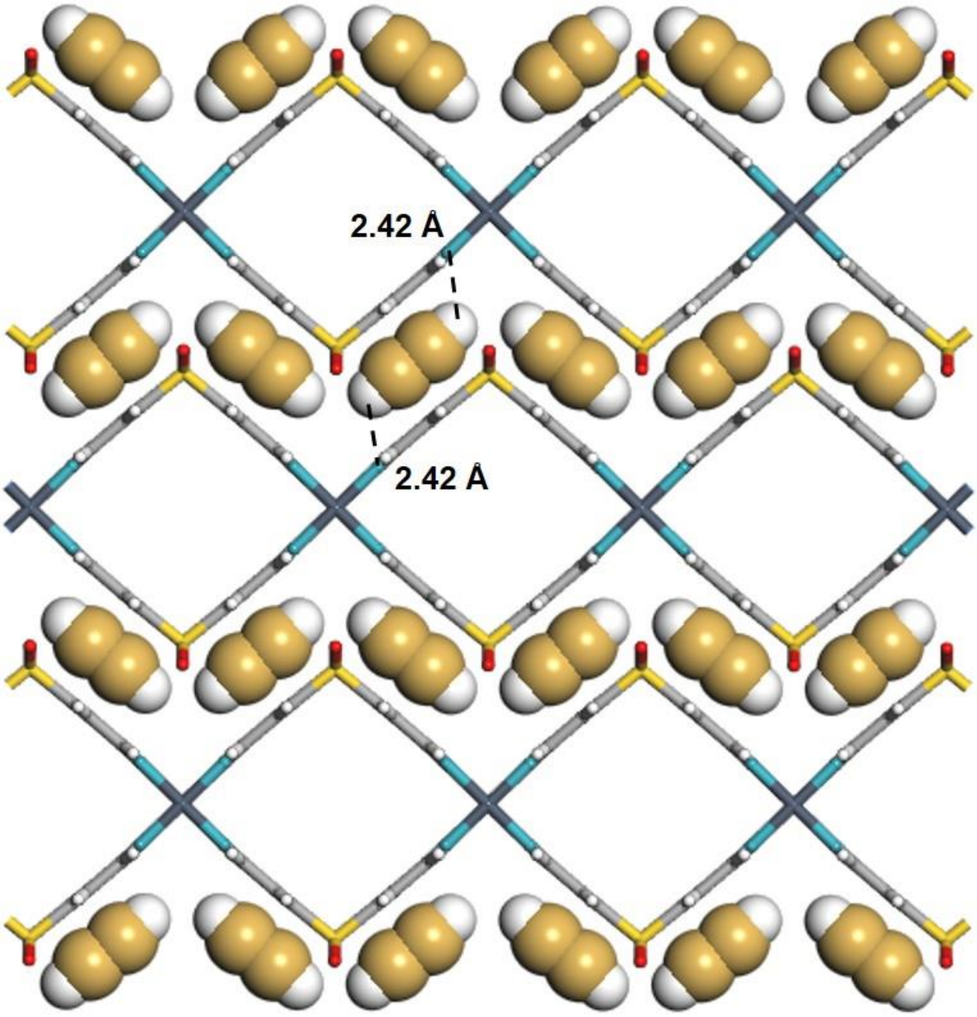

Supplementary Figure 29. The crystal structure of ZUL-200 under C_2H_2 (C_2H_2@ZUL-200) at ultralow pressure (Color code: F, teal; Nb, plain blue; C, gray; H, white; N, sky blue; S, yellow; O, red; Cu, blue; C (in C_2H_2), golden).

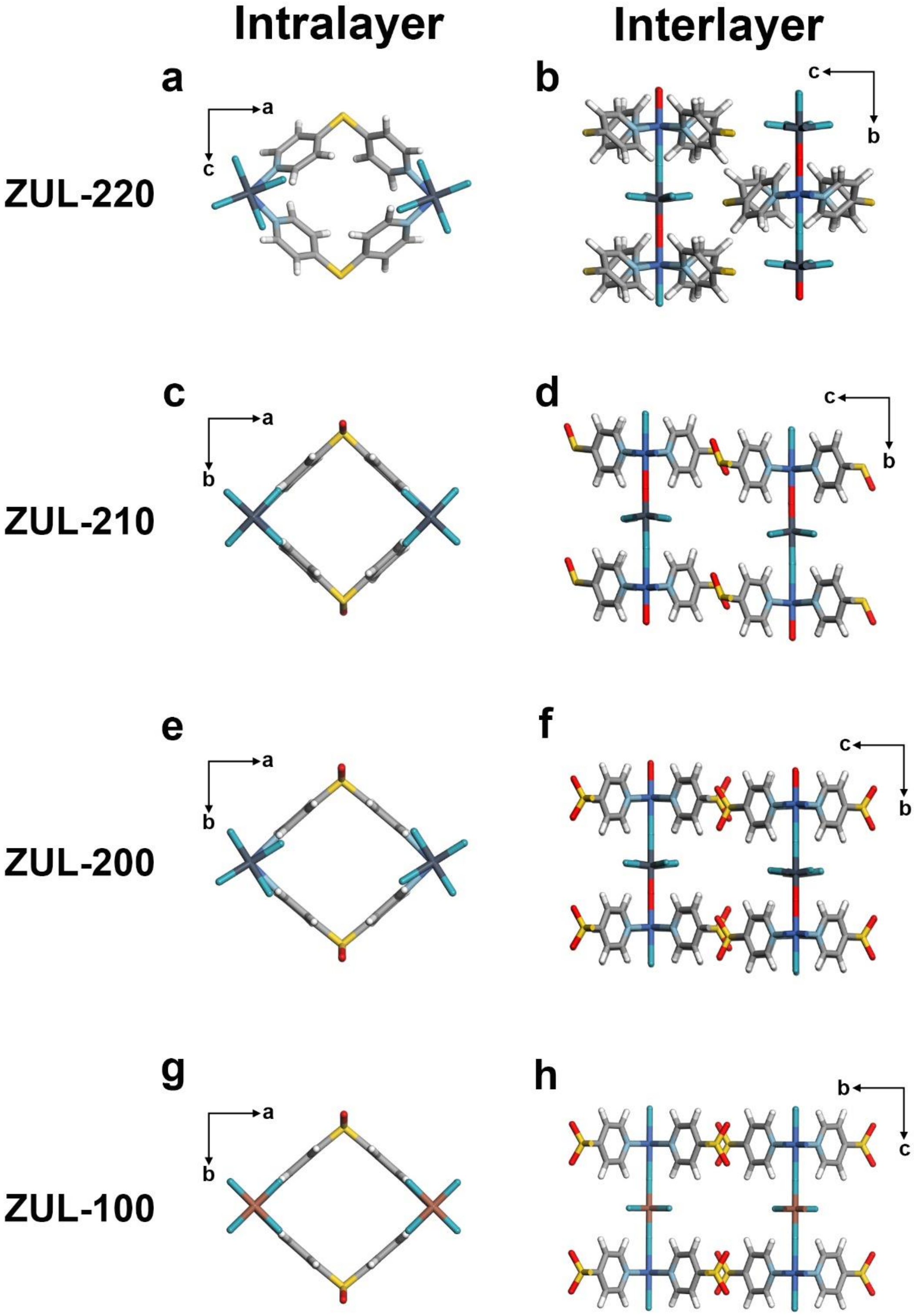

Supplementary Figure 30. DFT optimized geometry of MOFs (Color code: F, teal; Nb, plain blue; Ti, brown; C, gray; H, white; N, sky blue; S, yellow; O, red; Cu, blue).

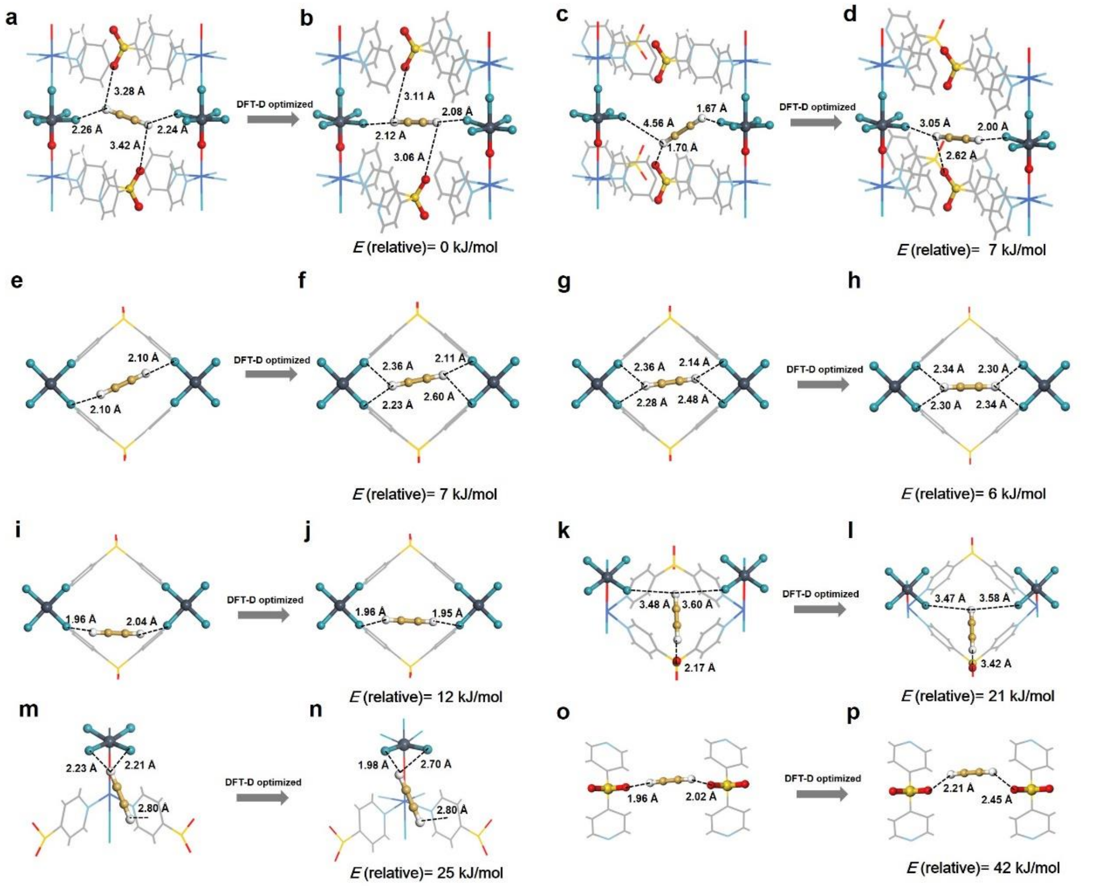

Supplementary Figure 31. Initial configurations C_2H_2 adsorption sites of ZUL-200 and DFT-D optimized configurations with their relative energy (the difference with the energy of configuration *b*) (Color code: F, teal; Nb, plain blue; C (in framework), gray; H, white; N, sky blue; S, yellow; O, red; Cu, blue; C (in C_2H_2), golden).

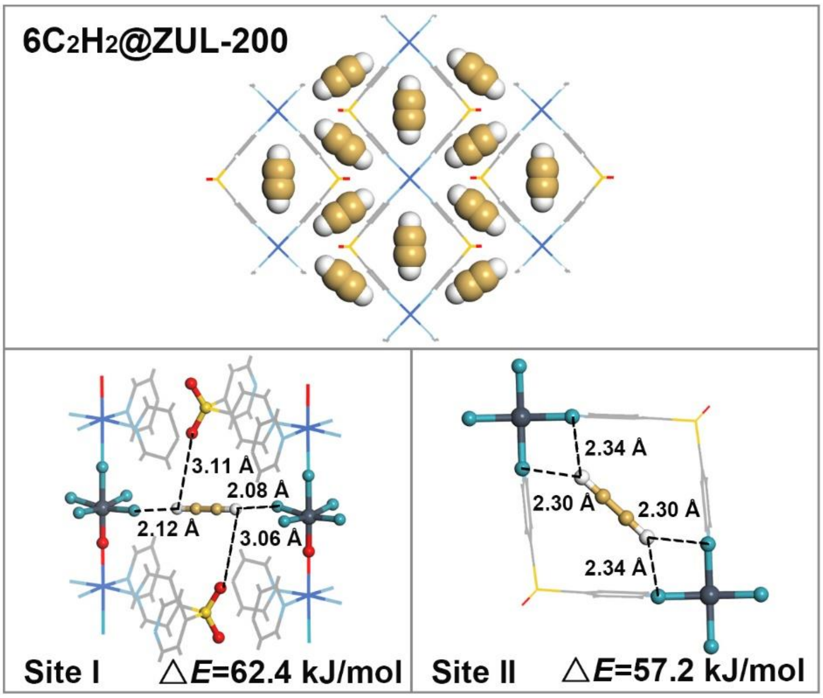

Supplementary Figure 32. DFT-D optimized C_2H_2 adsorption sites of $6C_2H_2$@ZUL-200 (Color code: F, teal; Nb, plain blue; C (in framework), gray; H, white; N, sky blue; S, yellow; O, red; Cu, blue; C (in C_2H_2), golden).

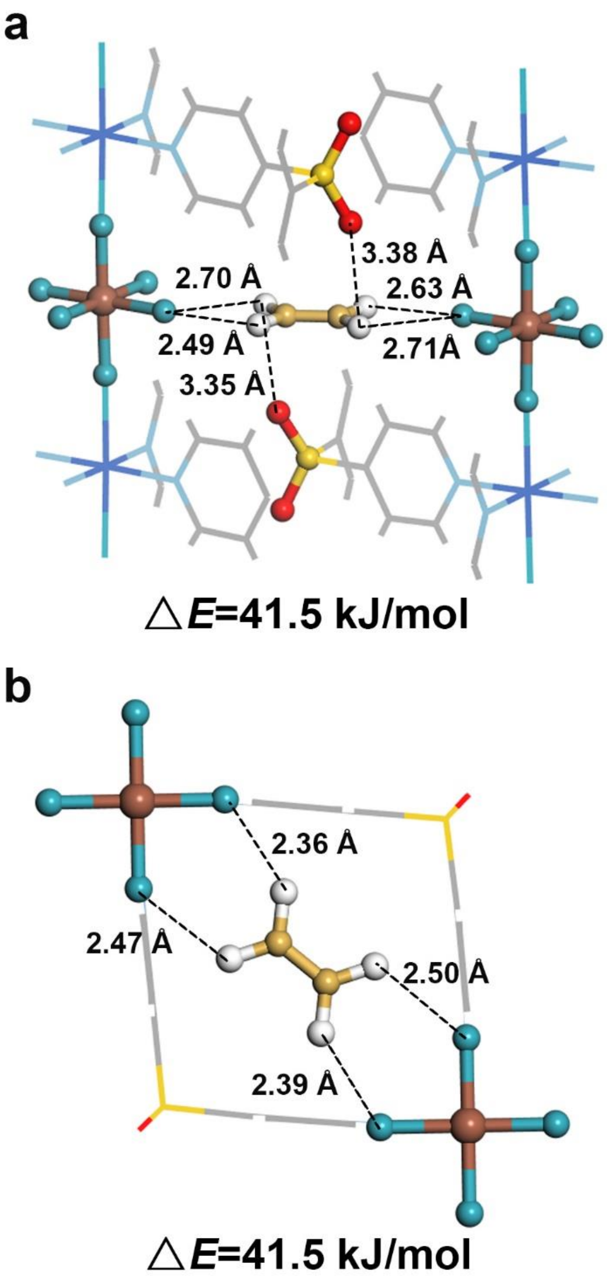

Supplementary Figure 33. DFT-D optimized C_2H_4 adsorption sites of ZUL-100 (Color code: F, teal; Ti, brown; C (in framework), gray; H, white; N, sky blue; S, yellow; O, red; Cu, blue; C (in C_2H_4), golden).

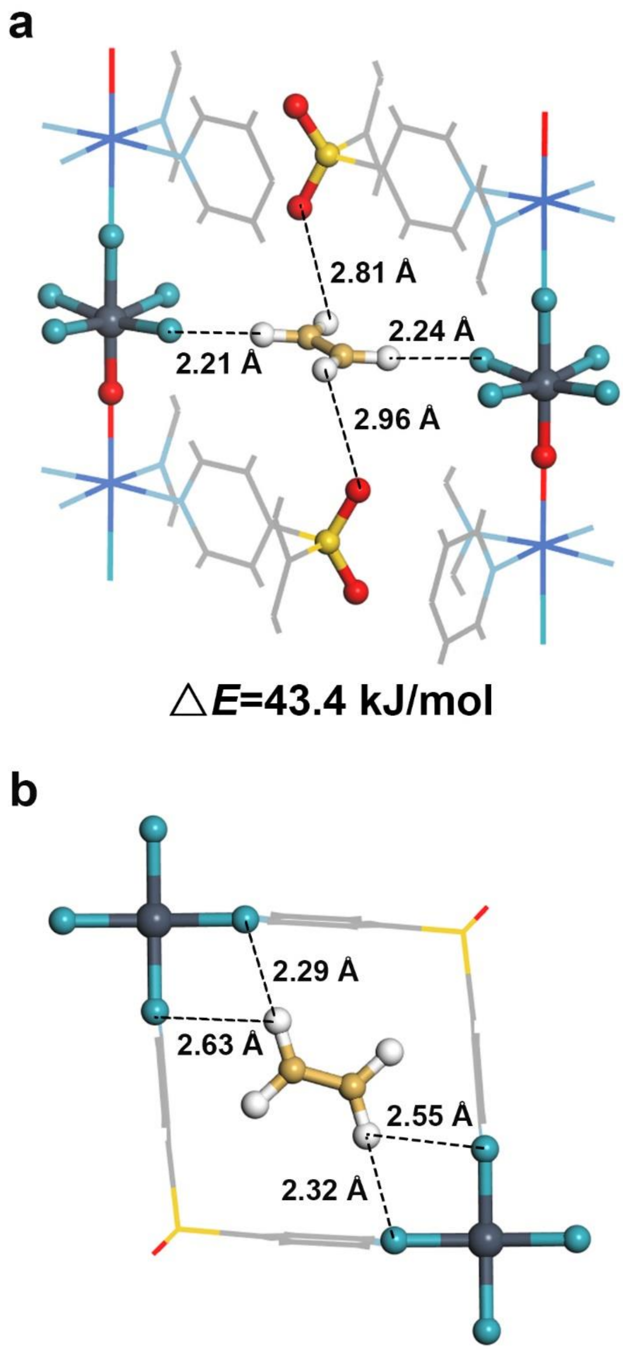

Supplementary Figure 34. DFT-D optimized C_2H_4 adsorption sites of ZUL-200 (Color code: F, teal; Nb, plain blue; C (in framework), gray; H, white; N, sky blue; S, yellow; O, red; Cu, blue; C (in C_2H_4), golden).

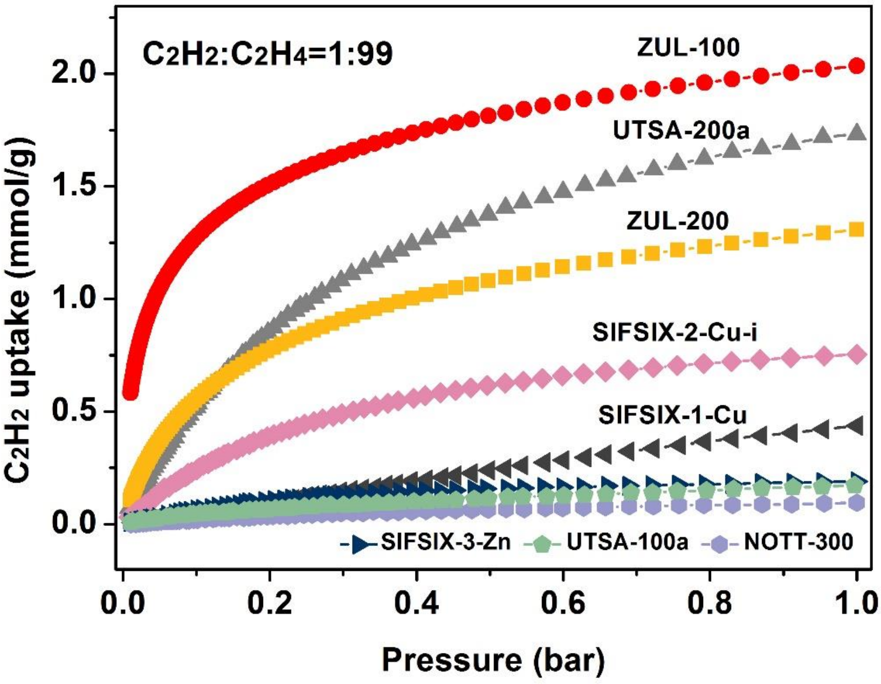

Supplementary Figure 35. Simulated C_2H_2 uptake of C_2H_2/C_2H_4 (1/99) mixture on various MOF materials at 298 K

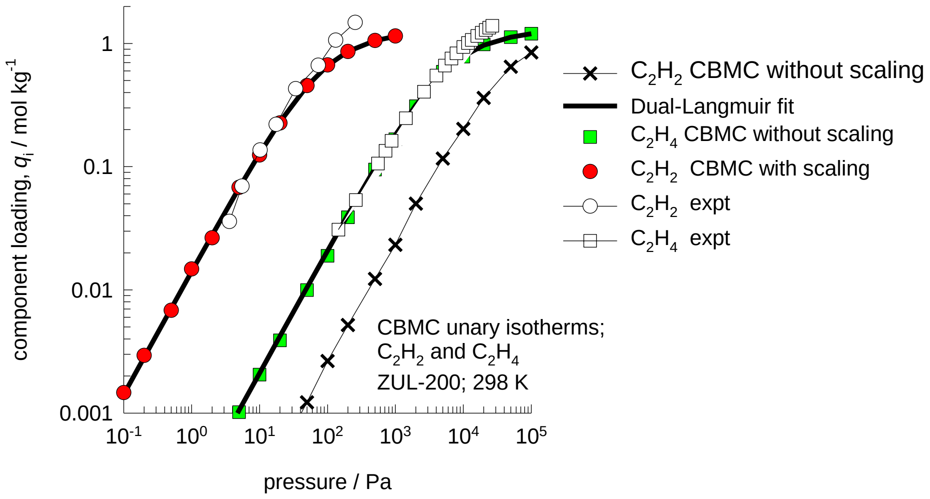

Supplementary Figure 36. Comparing CBMC simulations of unary isotherms for C_2H_2 (crosses: without scaling; red circles with scaling) and C_2H_4 (without scaling) in ZUL-200 at 298 K with experimental data on unary isotherms in the Henry regime.

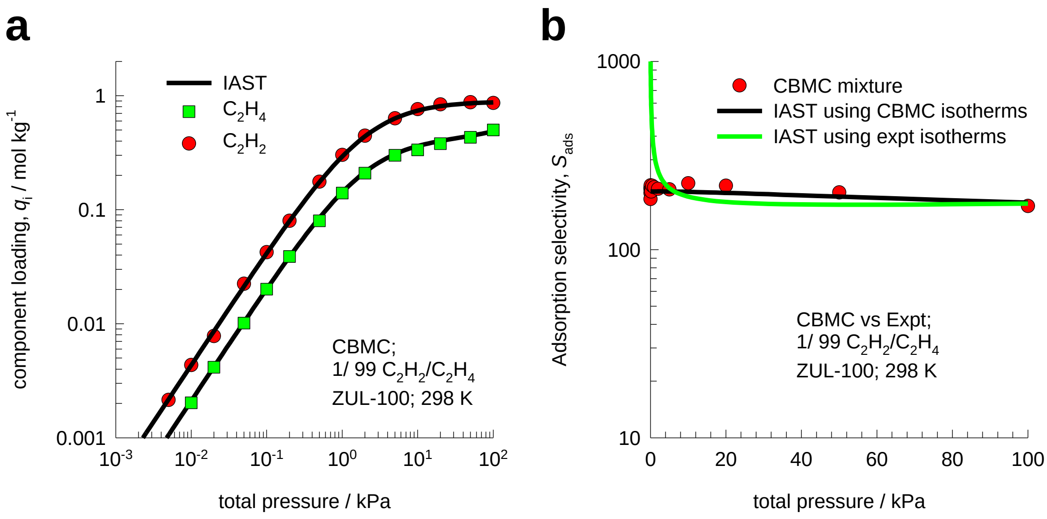

Supplementary Figure 37. (a) CBMC simulations for adsorption of 1/99 C_2H_2/C_2H_4 mixtures in ZUL-100 at 298 K. The continuous solid lines are IAST calculations of adsorption equilibrium using the dual-Langmuir fits of unary isotherms. (b) CBMC simulations for adsorption selectivity of 1/99 C_2H_2/C_2H_4 mixtures in ZUL-100 at 298 K. The continuous solid lines are IAST calculations using unary isotherms fitted with CBMC data and experimental data.

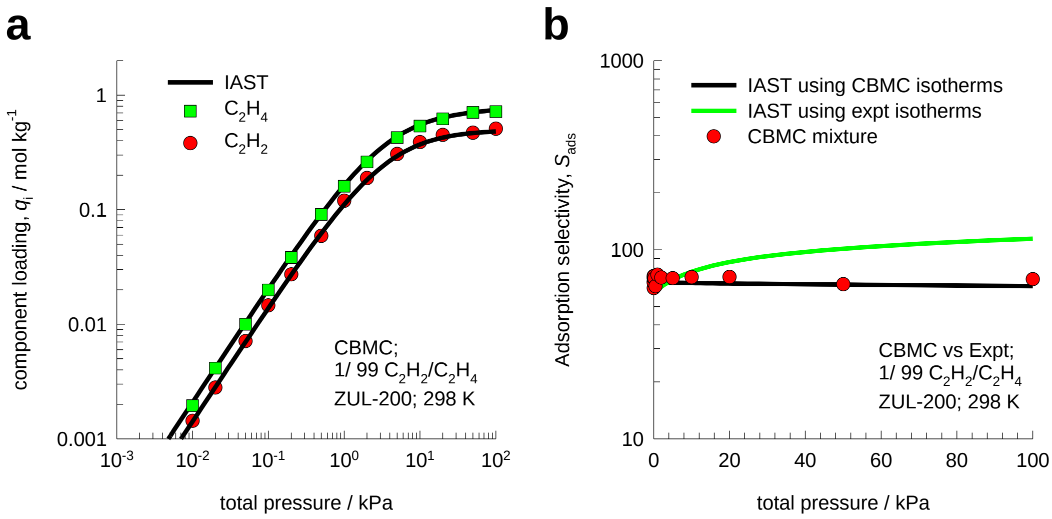

Supplementary Figure 38. (a) CBMC simulations for adsorption of 1/99 C_2H_2/C_2H_4 mixtures in ZUL-200 at 298 K. The continuous solid lines are IAST calculations of adsorption equilibrium using the dual-Langmuir fits of unary isotherms. (b) CBMC simulations for adsorption selectivity of 1/99 C_2H_2/C_2H_4 mixtures in ZUL-200 at 298 K. The continuous solid lines are IAST calculations using unary isotherms fitted with CBMC data and experimental data.

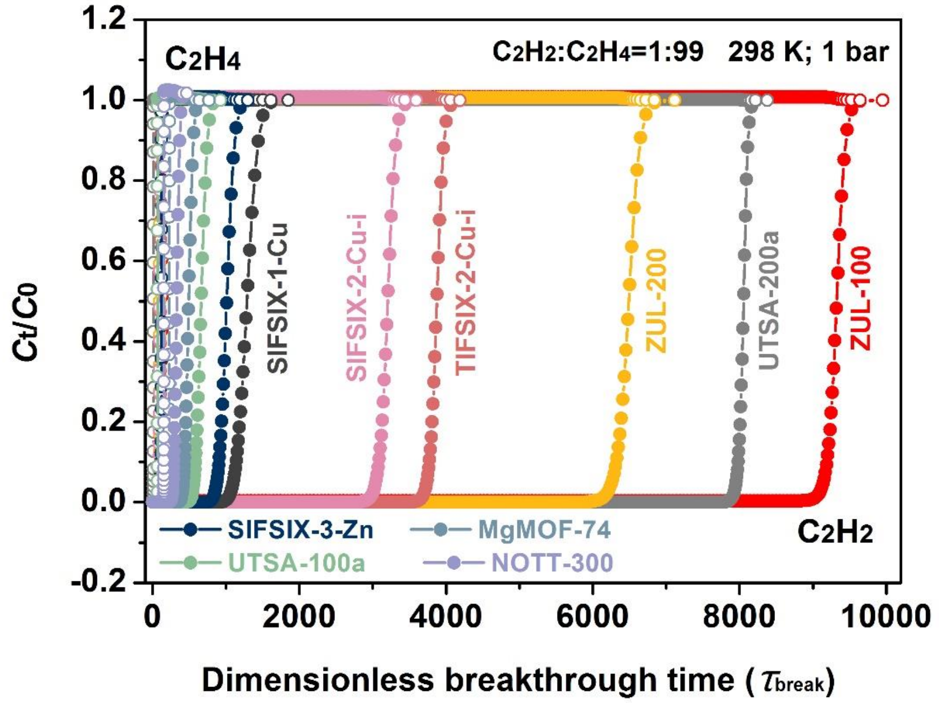

Supplementary Figure 39. Simulated column breakthrough curves for C_2H_2/C_2H_4 separation with respect to various MOF materials as indicated.

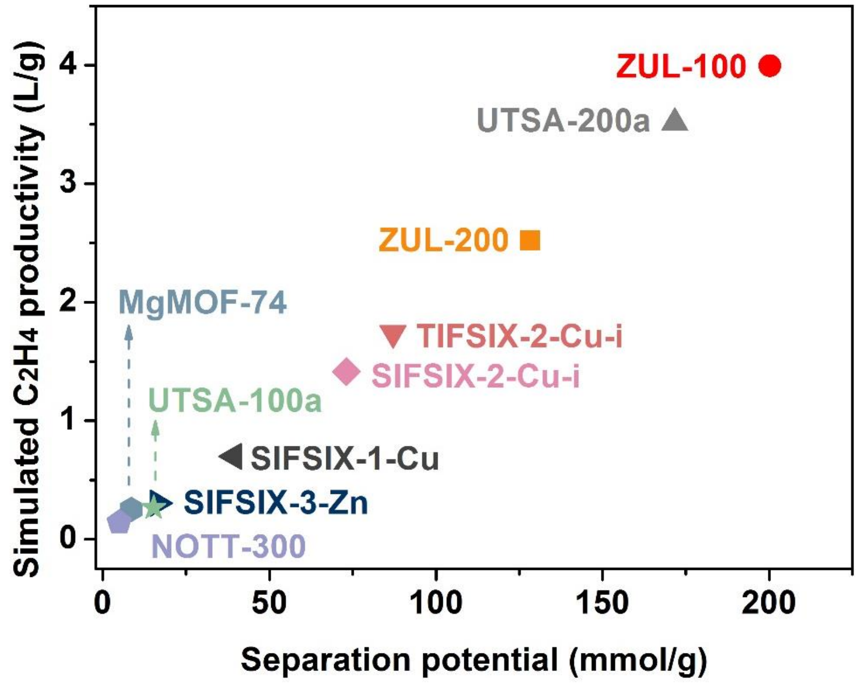

Supplementary Figure 40. Plots of the productivity of purified C_2H_4 (<40 ppm C_2H_2) from transient breakthrough simulation as a function of separation potential from IAST calculation for C_2H_2/C_2H_4 (1/99) mixture for various MOF materials as indicated.

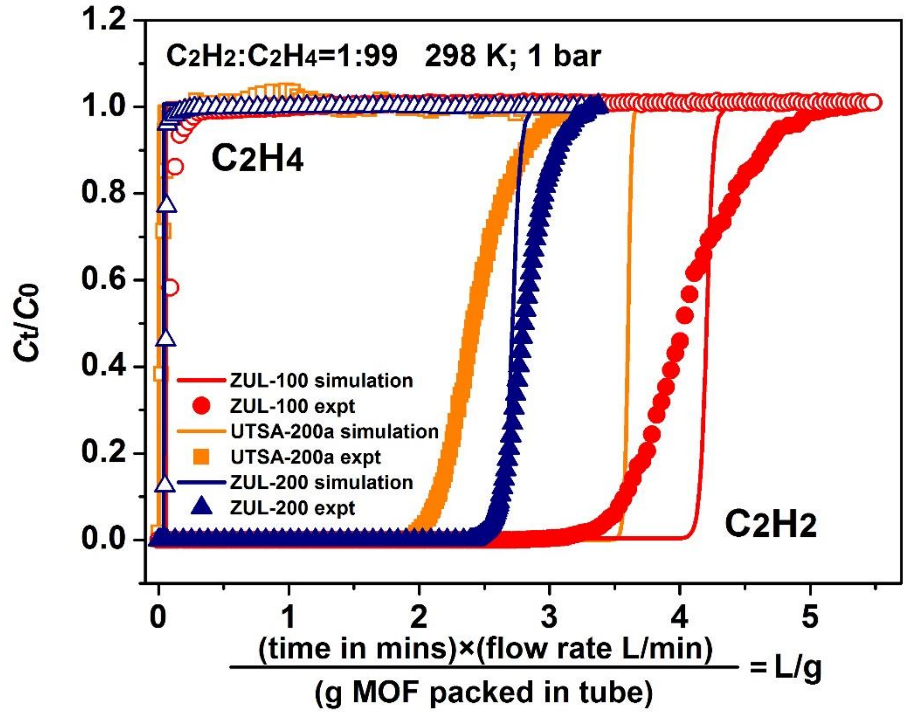

Supplementary Figure 41. Experimental breakthrough data and simulated transient breakthrough of C_2H_2:C_2H_4=1:99 mixture through fixed bed adsorbers packed with ZUL-100, ZUL-200 and UTSA-200 operating at 298 K and 1bar.

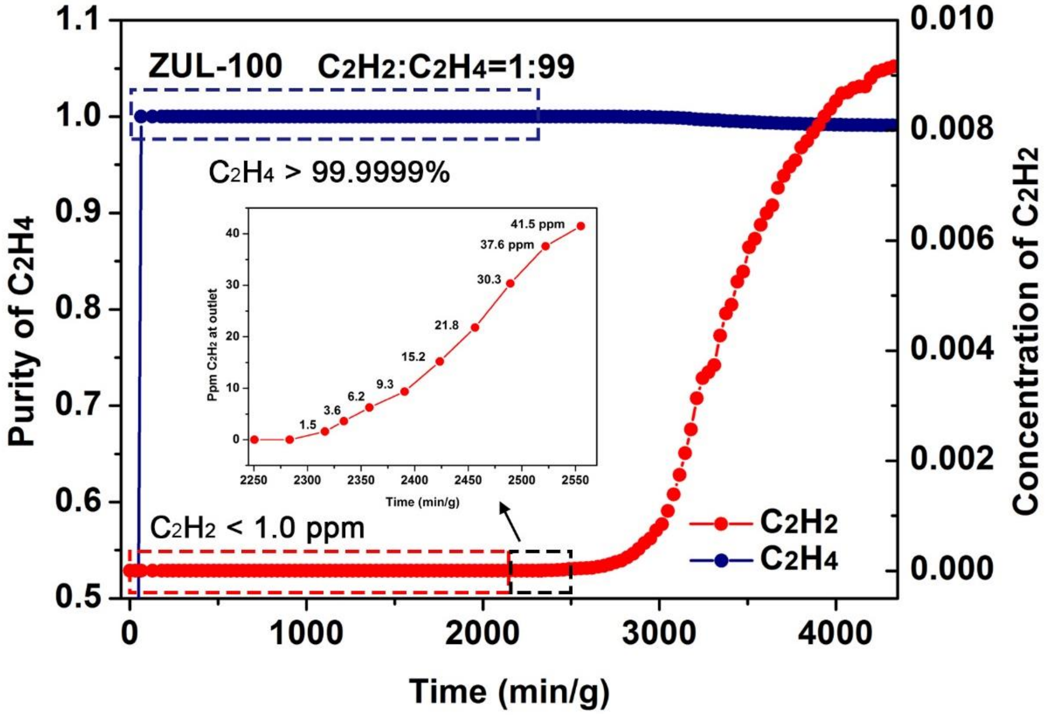

Supplementary Figure 42. The concentration of C_2H_2 and the purity of C_2H_4 in the outlet gas of the adsorber. The inserted figure shows the C_2H_2 content in the outlet gas in ppm. Experimental breakthrough was conducted on a stainless steel column packed with ZUL-100 (4.6×50 mm) with C_2H_2/C_2H_4 (1/99) mixtures as feed gas at 1.25 ml/min, 298 K and 1 bar.

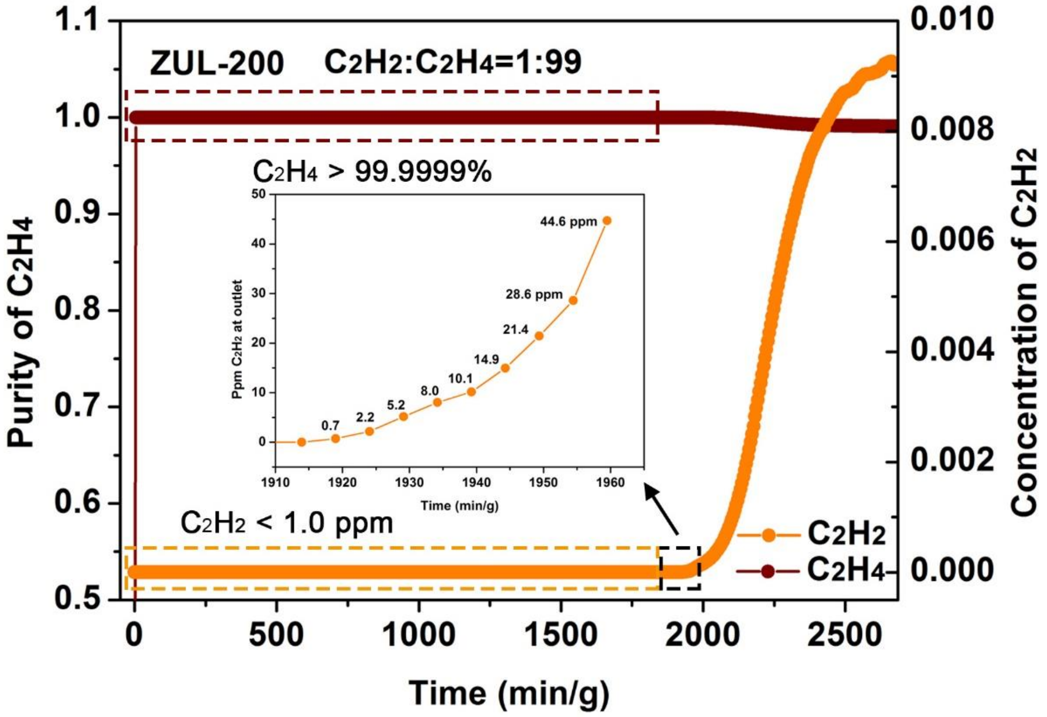

Supplementary Figure 43. The concentration of C_2H_2 and the purity of C_2H_4 in the outlet gas of the adsorber. The inserted figure shows the C_2H_2 content in the outlet gas in ppm. Experimental breakthrough was conducted on a stainless steel column packed with ZUL-200 (4.6×50 mm) with C_2H_2/C_2H_4 (1/99) mixtures as feed gas at 1.25 ml/min, 298 K and 1 bar.

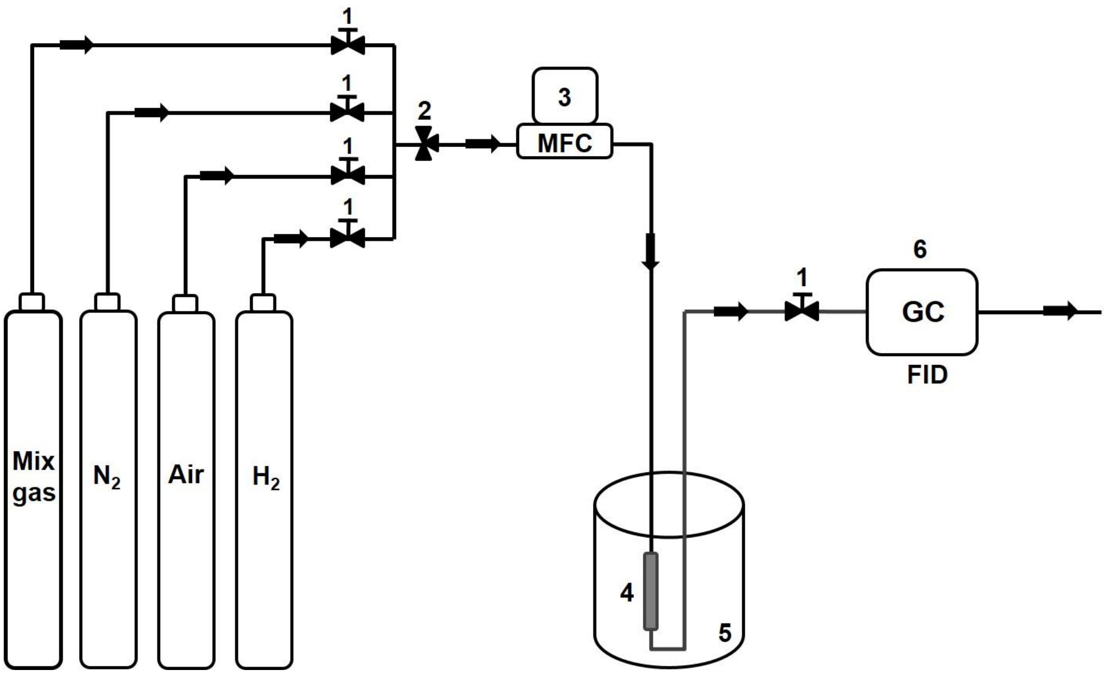

Supplementary Figure 44. The schematic breakthrough experiments device. 1. Valve 2. 3-way valve 3. Mass flow controller 4. Colum 5. Mantle heater 6. Gas chromatography with FID monitor

Supplementary Table 1-13

Supplementary Table 1. Crystal structure data and refinement condition for as-synthesized ZUL-220

Unit cell parameters	
Formula sum	$C_{20} H_{16} N_4 O F_5 S_2$ Cu Nb
Formula weight	643.94
Crystal system	Orthorhombic
Space-group	Ibam
Cell parameters	a=19.3295(17) Å b=9.8077(9) Å c=16.4662(15) Å
Cell ratio	a=b=c=90°
Cell volume	3121.6(5) $Å^3$
Z	4
Calc. density	1.37016 g/cm^3

Supplementary Table 2. Crystal structure data and refinement condition for activated ZUL-220

Unit cell parameters	
Formula sum	$C_{20} H_{16} N_4 O F_5 S_2$ Cu Nb
Formula weight	643.94
Crystal system	Monoclinic
Space-group	P 2
Cell parameters	a=9.8642(9) Å b=8.2564(6) Å c=16.8333(13) Å
Cell ratio	a=90° b=97.045° c=90°
Cell volume	1360.60(19) $Å^3$
Z	2
Calc. density	1.57178 g/cm^3

Supplementary Table 3. Crystal structure data and refinement condition for as-synthesized ZUL-210

Unit cell parameters	
Formula sum	$C_{20} H_{16} N_4 O_3 F_5 S_2$ Cu Nb
Formula weight	675.94
Crystal system	Orthorhombic
Space-group	Cmmm
Cell parameters	a=9.6213(8) Å b=19.0396(17) Å c=8.6179(8) Å
Cell ratio	a=b=c=90°
Cell volume	1578.7(2) $Å^3$
Z	2
Calc. density	1.42197 g/cm^3

Supplementary Table 4. Crystal structure data and refinement condition for activated ZUL-210

Unit cell parameters	
Formula sum	C_{20} H_{16} N_4 O_3 F_5 S_2 Cu Nb
Formula weight	675.94
Crystal system	Orthorhombic
Space-group	Cmmm
Cell parameters	a=9.789(3) Å b=19.102(7) Å c=8.381(2) Å
Cell ratio	a=b=c=90°
Cell volume	1567.1(8) Å^3
Z	2
Calc. density	1.43242 g/cm^3

Supplementary Table 5. Crystal structure data and refinement condition for as-synthesized ZUL-200

Unit cell parameters	
Formula sum	C_{20} H_{16} N_4 O_5 F_5 S_2 Cu Nb
Formula weight	707.94
Crystal system	Orthorhombic
Space-group	Cmmm
Cell parameters	a=9.8421(5) Å b=19.0521(10) Å c=8.7095(5) Å
Cell ratio	a=b=c=90°
Cell volume	1633.14(15) Å^3
Z	2
Calc. density	1.43962 g/cm^3

Supplementary Table 6. Crystal structure data and refinement condition for activated ZUL-200

Unit cell parameters	
Formula sum	C_{20} H_{16} N_4 O_5 F_5 S_2 Cu Nb
Formula weight	707.94
Crystal system	Orthorhombic
Space-group	Pmmn
Cell parameters	a=9.8447(15) Å b=19.122(3) Å c=8.5540(15) Å
Cell ratio	a=b=c=90°
Cell volume	1610.3(5) Å^3
Z	2
Calc. density	1.46005 g/cm^3

Supplementary Table 7. Crystal structure data and refinement condition for as-synthesized ZUL-100

Unit cell parameters	
Formula sum	C_{20} H_{16} N_4 O_4 F_6 S_2 Cu Ti
Formula weight	665.93
Crystal system	Orthorhombic
Space-group	Cmmm
Cell parameters	a=9.8088(16) Å b=19.283(3) Å c=8.6328(12) Å
Cell ratio	a=b=c=90°
Cell volume	1632.83(4) $Å^3$
Z	2
Calc. density	1.35445 g/cm^3

Supplementary Table 8. Crystal structure data and refinement condition for C_2H_2@ZUL-200

Unit cell parameters	
Formula sum	C_{24} H_{20} N_4 O_5 F_5 S_2 Cu Nb
Formula weight	760.01
Crystal system	Orthorhombic
Space-group	Cmmm
Cell parameters	a=9.8345(6) Å b=19.1172(15) Å c=8.5600(5) Å
Cell ratio	a=b=c=90°
Cell volume	1609.35(19) $Å^3$
Z	2
Calc. density	1.56837 g/cm^3

Supplementary Table 9. Langmuir-Freundlich parameter fits for C_2H_2 and C_2H_4 in ZUL-100 at 298 K

	Site A			Site B		
	$q_{A,sat}$ (mmol/g)	b_A (Pa^{-1})	v_A	$q_{B,sat}$ (mmol/g)	b_B (Pa^{-1})	v_B
C_2H_2	3.8	6.663E-03	0.43	3.54	5.008E-02	0.57
C_2H_4	1.6	1.689E-05	1	1.8	2.454E-04	1

Supplementary Table 10. Langmuir-Freundlich parameter fits for C_2H_2 and C_2H_4 in ZUL-200 at 298 K

	Site A			Site B		
	$q_{A,sat}$ (mmol/g)	b_A (Pa^{-1})	v_A	$q_{B,sat}$ (mmol/g)	b_B (Pa^{-1})	v_B
C_2H_2	2.7	4.479E-03	1	2.2	6.867E-05	1
C_2H_4	1.25	1.290E-05	1	1.35	1.421E-04	1

Supplementary Table 11. The C_2H_4 productivity calculation parameters in breakthrough experiment for C_2H_2/C_2H_4 (1/99, v/v) mixture on various MOF materials

	F (ml/min)	*t* (min/g) (C_2H_2 < 40 ppm)	Ref.
ZUL-100	1.25	2546	This work
ZUL-200	1.25	1958	This work
UTSA-200a	1.25	1650	1
TIFSIX-2-Cu-i	10	185.9	3
ZU-33	1.25	1625	4
SIFSIX-2-Cu-i	1.25	952	5
SIFSIX-1-Cu	1.25	330	5
SIFSIX-3-Zn	1.25	56	5
UTSA-100a	2	15	8

Supplementary Table 12. The volumetric uptake of C_2H_2 and C_2H_4 in various MOFs including ZUL-100 and ZUL-200

	C_2H_2 uptake at 0.01 bar (mmol/ml), 298 K	C_2H_2 uptake at 1.0 bar (mmol/ml), 298 K	C_2H_4 uptake at 1.0 bar (mmol/ml), 298 K
ZUL-100	4.01	7.19	3.74
ZUL-200	3.26	6.85	2.91
UTSA-200a[1]	2.59	5.18	0.89
NKMOF-1-Ni[2]	3.03	4.77	3.70
TIFSIX-2-Cu-i[3]	2.42	5.84	3.56
ZU-33[4]	2.75	5.31	0.98
SIFSIX-2-Cu-i[5]	2.02	5.01	2.73
SIFSIX-3-Zn[5]	1.34	5.73	3.53
SIFSIX-1-Cu[5]	0.39	7.34	3.53
UTSA-100a[8]	0.91[a]	4.89[a]	1.90[a]
NOTT-300[9]	0.19[b]	6.73[b]	4.55[b]
MgMOF-74[15]	2.01[a]	6.80[a]	6.04[a]

[a] At a temperature of 296 K

[b] At a temperature of 293 K

Supplementary Table 13. Comparison of the adsorption uptakes, Q_{st} data, selectivity of C_2H_2/C_2H_4 for various MOFs including ZUL-100 and ZUL-200.

	Dimension	S_{BET}[a] (m^2/g, BET)	Pore size (Å)	C_2H_2 uptake at 0.01 bar (mmol/g), 298 K	C_2H_2 uptake at 1.0 bar (mmol/g), 298 K	C_2H_4 uptake at 1.0 bar (mmol/g), 298 K	Selectivities for C_2H_2 / C_2H_4 at 1/99 mixtures 1 bar	Q_{st} (C_2H_2,KJ/mol)[b]
ZUL-100	2D	548	3.6×4.1<br>3.1×4.4	2.96	5.31	2.76	175	65.3
ZUL-200	2D	471	3.6×4.1<br>3.3×4.4	2.23	4.69	1.99	114	57.6
UTSA-200a[1]	3D	612	3.4×3.4	1.85	3.65	0.63	6320	40
NKMOF-1-Ni[2]	3D	380	5.75×5.75	1.73	2.72	2.11	51.65	18.8/54[g]
TIFSIX-2-Cu-i[3]	3D	685	5.5	1.70	4.10	2.50	55	46.3
ZU-33[4]	3D	424	3.0	1.66	3.21	0.59	1100	43.6
SIFSIX-2-Cu-i[5]	3D	503	5.2×5.2	1.62	4.02	2.19	44.54	41.9
SIFSIX-3-Zn[5]	3D	250	4.2×4.2	0.85	3.64	2.24	8.82	21/31[g]
SIFSIX-3-Ni[5]	3D	368	4.2×4.2	/	3.3	1.75	5.03	30.5
SIFSIX-2-Cu[5]	3D	1881	10.5×10.5	/	5.38	2.02	6	26.3
SIFSIX-1-Cu[5]	3D	1178	8.0×8.0	0.45	8.5	4.11	10.63	30/37[g]
UTSA-300a[6]	2D	311	2.4×3.3	0	3.41	0.04	/	57.6
NKMOF-1-Cu[2]	3D	280	5.75×5.75	/	2.28	/	/	/
M'MOF-3a[7]	3D	110	3.4×4.8	0.19	1.9[c]	0.4[c]	24.03	25
UTSA-100a[8]	3D	970	4.3×4.3	0.80[c]	4.27[c]	1.66[c]	10.72	22
NOTT-300[9]	3D	1370	6.5×6.5	0.18	6.34[d]	4.28[d]	2.17	32
FeMOF-74[10]	3D	1350	11×11	/	6.8[e]	6.1[e]	2.08	46
ZJU-40a[11]	3D	2858	10.2×10.2;<br>9.6×22.3	0.65	9.64	/	/	34.5
FJI-H8[12]	3D	2025	15; 8; 12	0.7[f]	10[f]	/	/	32.0

HKUST-1[13]	3D	1401	9×9	1.1[f]	8.97[f]	/	/	30.4
ZJU-5a[14]	3D	2823	10.5; 9.5×22.5	0.35	8.62	/	/	35.8
MgMOF-74[15]	3D	1495	12	2.21[c]	7.48[c]	6.65[c]	2.0	41

[a] BET surface calculated from N_2 isotherms at 77 K.

[b] Q_{st} values at low surface coverage.

[c] At a temperature of 296 K

[d] At a temperature of 293 K

[e] At a temperature of 318 K

[f] At a temperature of 295 K

[g] The highest Q_{st} values at various surface coverage.

Supplementary Table 14. Lennard-Jones parameters for host atoms in ZUL-100 and ZUL-200.

atom	σ_{host} (Å)	$\frac{\varepsilon_{host}}{k_B}$ (K)	Literature source
Cu	3.1137	2.5164	UFF[16]
F	3.0932	36.4872	DREIDING[17]
N	3.2626	38.9532	DREIDING[17]
S	3.5903	173.1253	DREIDING[17]
Nb	2.8197	29.6930	UFF[16]
Ti	2.8286	8.5556	UFF[16]
C	3.4730	47.8611	DREIDING[17]
O	3.0332	48.1631	DREIDING[17]
H	2.8464	7.6497	DREIDING[17]

Supplementary Table 15. Lennard-Jones parameters for guest pseudo-atoms

(pseudo-) atom	σ_{guest} (Å)	$\frac{\varepsilon_{guest}}{k_B}$ (K)	Literature source
-CH	3.8	57.8782776	Gautam et al.[18]
$-CH_2$	3.68	92.5	Ban et al.[19]

Supplementary Table 16. Lennard-Jones parameters for the guest – host (F atoms) interactions.

(pseudo-) atom	host atom	$\sigma_{guest-host}$ (Å)	$\frac{\varepsilon_{guest-host}}{k_B}$ (K)
-CH	F	$\frac{(3.8+3.0932)}{2}=3.4466$	$6\times\sqrt{57.87828\times 36.4872} = 275.727$ (This is fitted value)[a]
$-CH_2$	F	$\frac{(3.68+3.0932)}{2}=3.3866$	$\sqrt{92.50\times 36.4872}=58.09529$

[a] The scaling factor 6 is artificial and cannot be used for any other simulations except fitting the experimental data to support the reliability of the IAST calculations in this work.

Supplementary References

1. Li, B. et al. An ideal molecular sieve for acetylene removal from ethylene with record selectivity and productivity. *Adv. Mater.* **29,** 1704210-1704216 (2017).

2. Peng, Y. et al. Robust Ultramicroporous metal-organic frameworks with benchmark affinity for acetylene. *Angew. Chem. Int. Ed.* **57**, 10971-10975 (2018).

3. Bajpai, A. et al. The effect of centred versus offset interpenetration on C_2H_2 sorption in hybrid ultramicroporous materials. *Chem. Commun.* **53,** 11592-11595 (2017).

4. Zhang, Z. et al. Hexafluorogermanate (GeFSIX) anion-functionalized hybrid ultramicroporous materials for efficiently trapping acetylene from ethylene. *Ind. Eng. Chem. Res.* **57**, 7266-7274 (2018).

5. Cui, X. et al. Pore chemistry and size control in hybrid porous materials for acetylene capture from ethylene. *Science* **353,** 141-144 (2016).

6. Lin, R. et al. Optimized separation of acetylene from carbon dioxide and ethylene in a microporous material. *J. Am. Chem. Soc.* **139,** 8022-8028 (2017).

7. Das, M. C. et al. Interplay of metalloligand and organic ligand to tune micropores within isostructural mixed-metal organic frameworks (M'MOFs) for their highly selective separation of chiral and achiral small molecules. *J. Am. Chem. Soc.* **134,** 8703-8710 (2012).

8. Hu, T. et al. Microporous metal–organic framework with dual functionalities for highly efficient removal of acetylene from ethylene/acetylene mixtures. *Nat. Commun.* **6,** 7328-7335 (2015).

9. Yang, S. et al. Supramolecular binding and separation of hydrocarbons within a functionalized porous metal–organic framework. *Nat. Chem.* **7,** 121-129 (2014).

10. Bloch, E. D. et al. Hydrocarbon separations in a metal-organic framework with open Iron(II) coordination sites. *Science* **335,** 1606-1610 (2012).

11. Wen, H. et al. A microporous metal–organic framework with Lewis basic nitrogen sites for high C_2H_2 storage and significantly enhanced C_2H_2/CO_2 separation at ambient conditions. *Inorg. Chem.* **55,** 7214-7218 (2016).

12. Pang, J. et al. A porous metal-organic framework with ultrahigh acetylene uptake capacity under ambient conditions. *Nat. Commun.* **6,** 7575-7581 (2015).

13. Xiang, S., Zhou, W., Gallegos, J. M., Liu, Y. & Chen, B. Exceptionally high acetylene uptake in a microporous metal-organic framework with open metal sites. *J. Am. Chem. Soc.* **131,** 12415-12419 (2009).

14. Rao, X. et al. A microporous metal–organic framework with both open metal and Lewis basic pyridyl sites for high C_2H_2 and CH_4 storage at room temperature. *Chem. Commun.* **49,** 6719-6721 (2013).

15. He, Y., Krishna, R. & Chen, B. Metal–organic frameworks with potential for energy-efficient adsorptive separation of light hydrocarbons. *Energy Environ. Sci.* **5,** 9107-9120 (2012).

16. Rappé, A. K. et al. UFF, A full periodic table force field for molecular mechanics and molecular dynamics simulations. *J. Am. Chem. Soc.* **114,** 10024-10035 (1992).

17. Mayo, S. L., Olafson, B. D. & Goddard, W. A. DREIDING: A generic force field for molecular simulations. *J. Phys. Chem.* **94,** 8897-8909 (1990).

18. Gautam, S. et al. Diffusion of acetylene inside Na-Y zeolite: Molecular dynamics simulation studies. *Phys. Rev. E* **74,** 041202 (2006).

19. Ban, S. et al. Adsorption selectivity of benzene and propene mixtures for various zeolites. *J. Phys. Chem. C* **111,** 17241-17248 (2007).
